# Regulation of Shade Avoidance Under Low‐Blue‐Light by MTA in Soybean

**DOI:** 10.1002/advs.202410334

**Published:** 2024-12-12

**Authors:** Liya Zhang, Jun Liu, Jiaqi Chen, Yanyan Zhang, Chao Qin, Xiangguang Lyu, Zhuang Li, Ronghuan Ji, Bin Liu, Hongyu Li, Tao Zhao

**Affiliations:** ^1^ State Key Laboratory of Crop Gene Resources and Breeding Key Laboratory of Soybean Biology (Beijing) (MARA) Institute of Crop Sciences Chinese Academy of Agricultural Sciences Beijing 100081 China

**Keywords:** blue light, MTA, N6‐methyladenosine (m6A), shade avoidance, soybean

## Abstract

Under low blue light (LBL) conditions, soybean exhibits classic shade avoidance syndrome (SAS) with exaggerated stem elongation (ESE), leading to lodging and yield reduction in dense farming. Recently, mRNA modification by N6‐methyladenosine (m6A) has emerged as a crucial epigenetic mechanism regulating plant biological processes; however, its impact on shade avoidance remains unexplored. In this study, the double mutants, *gmmtas*, that are impaired in two m^6^A writer genes, *GmMTAa* and *GmMTAb* that encode m^6^A methyltransferases or m^6^A writers are generated. It is found that the *gmmtas* mutants showed a substantial reduction of m6A levels, a dwarf phenotype, and a diminished sensitivity to LBL. Further investigation of the *gmmtas* mutants demonstrates that *GmMTA* regulates shade avoidance response by altering the expression of *GmCRY1s*, *GmSPAs*, and *GmCOP1s*, resulting in increased accumulation of *GmSTFs* that are known to suppress the shad avoidance response in response to LBL in soybean. The findings reveal a novel molecular mechanism regulating shade resistance in soybean, providing insights into the epigenetic mechanisms of plant adaptation to changing light environments and paving the way for the development of shade‐tolerant soybean varieties.

## Graphical abstract

This study explores the role of N6‐methyladenosine (m6A) in shade avoidance in soybean. By generating double mutants impaired in m6A writer genes, researchers found that m6A regulates shade avoidance response and shade resistance by altering the expression of blue light signaling genes, offering insights into plant adaptation to changing light environments.

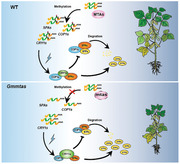

## 1 Introduction

The increasing demand for soybean worldwide, fueled by meat and soy‐based product consumption, population expansion, and biofuel policies, highlights the need to increase soybean production. While high‐density planting is a crucial approach for achieving this goal, it can cause shade avoidance syndrome (SAS) and exaggerated stem elongation (ESE), which diminish yield.^[^ ^1^, ^2^, ^3^ ^]^ Understanding regulatory mechanisms underlying soybean's shade avoidance response would facilitate breeding shade‐tolerant varieties with high yield potential.

Plants sense shade through photoreceptors such as phytochromes (PHYs) and cryptochromes (CRYs). In *Arabidopsis*, the PHYB photoreceptor mediates stem and petiole elongation in response to red‐light:far‐red light ratios. The absence of this receptor triggers shade avoidance responses. When activated by red light, PHYB translocates to the nucleus, where it interacts with phytochrome‐interacting factors (PIFs) to regulate growth‐related gene expression.^[^ ^4^ ^]^ CRYs sense low‐blue‐light (LBL), and interact directly with PIF4 and PIF5 to regulate shade avoidance and control plant growth.^[^ ^5^, ^6^ ^]^ A lack of CRY function mimics low red‐light:far‐red light (low R:FR) conditions, causing similar shade avoidance symptoms.

The growth and development of soybeans are highly sensitive to changes in the light environment, and factors such as light intensity, direction, and quality, as well as photoperiod duration, exert significant effects on yield traits such as plant height, branch count, main stem node number, pod count, stem strength, podding patterns, leaf senescence, flowering duration, and maturity period.^[^ ^2^, ^7^ ^]^ Specifically, the exposure of soybean to low R:FR ratios can markedly reduce leaf angle and stimulate stem elongation, while decreased blue light levels due to shading promote stem elongation and contribute to lodging.^[^ ^8^ ^]^ Studies have identified key regulatory genes involved in the blue light regulation pathway in soybean, and overexpression of *GmCRY1b* has been shown to confer strong shade tolerance. Conversely, the *Gmcry1s‐qm* quadruple mutant (*Gmcry1a1b1c1d*) exhibited stem elongation patterns similar to those of wild‐type (WT) soybean grown under LBL conditions.^[^ ^8^ ^]^ Mutations in genes such as *Gmcop1b* and *Gmspa3ab* lead to an increased abundance of STF1/2, resulting in reduced plant height and enhanced shade tolerance in soybean.^[^ ^9^, ^10^ ^]^

In recent years, the role of the N6‐methyladenosine (m6A) modification of mRNA in plant growth and development has attracted increasing attention.^[^ ^11^, ^12^, ^13^, ^14^ ^]^ Research has shown that m6A methylation is involved in regulating multiple biological processes such as plant embryonic development, stem cell fate, flower transformation, and responses to abiotic and biotic stress. For example, studies in *Arabidopsis* have revealed that the blue light receptor CRY1/CRY2 interacts with two different m6A methyltransferases, MTA and FIO1, to modulate the circadian clock and photomorphogenesis of plants.^[^ ^15^, ^16^, ^17^ ^]^ These findings offer insights into the regulatory mechanisms underlying the shade avoidance response in soybean plants, suggesting that m6A methylation may be a key modulator of this process.

The m6A methylation‐dependent regulation of gene expression is governed by a suite of enzymes known as writers, erasers, and readers, highlighting its adaptability in regulating gene expression.^[^ ^18^ ^]^ In *Arabidopsis*, the writers of m6A modification include proteins such as MTA, MTB, FIP37, VIRILIZER, and HAKAI, which catalyze the addition of m6A marks to mRNA molecules.^[^ ^19^, ^20^, ^21^, ^22^ ^]^ Conversely, eraser enzymes such as ALKBH9 and ALKBH10 are responsible for mRNA demethylation.^[^ ^23^, ^24^ ^]^ The recognition and functional interpretation of m6A ismediated by a range of m6A‐binding proteins, including YTH domain proteins and heterogeneous nuclear ribonucleoproteins (HNRNPs).^[^ ^25^, ^26^ ^]^ These proteins preferentially bind to specific sites within mRNA molecules, such as stop codons and 3' untranslated regions (3’UTRs),^[^ ^27^, ^28^ ^]^ suggesting that these proteins play regulatory roles in mRNA metabolism and translation.

The impact of m6A methylation on plant growth and development is becoming increasingly clear. In *Arabidopsis*, m6A modification has been shown to significantly influence various aspects of plant development, including embryogenesis, apical dominance, organ size, flowering time, leaf morphogenesis, and shoot apical meristem differentiation.^[^ ^20^, ^22^, ^29^, ^30^, ^31^ ^]^ In addition to *Arabidopsis*, m6A methylation has been implicated in the regulation of fruit ripening via the abscisic acid (ABA) pathway in strawberry plants and in the expansion and ripening of tomato fruit.^[^ ^32^, ^33^, ^34^ ^]^ Furthermore, in crops such as rice and potato, the modulation of m6A levels via human FTO‐like demethylases has led to significant increases in yield,^[^ ^35^ ^]^ highlighting the potential of this approach for improving crop productivity and addressing global food security challenges.

In this study, we identified a novel role for m6A methylation in modulating the mRNA expression of the *CRY1*, *SPA1*, and *COP1* genes, thereby regulating SAS and ESE in soybean. The knockout of GmMTAs resulted in reduced m6A levels in *GmCRY1s*, *GmSPAs*, and *GmCOP1s*, leading to altered expression of these genes. This indirectly influenced the transcription and protein levels of STF1/STF2. Notably, the strong line *Gmmtas‐dm‐4* exhibited an ideal shade resistance phenotype both under LBL conditions and in field experiments. Our findings reveal the critical role of GmMTA‐mediated m6A methylation in regulating LBL‐induced SAS in soybean and provide valuable insights for high‐density soybean breeding efforts.

## 2 Results

### 2.1 GmMTAs Mediate mRNA m6A Methylation in Soybean Plants

Soybean possesses two *MTA* genes, *GmMTAa* and *GmMTAb* (Figure S1a–c, Supporting Information), which display distinct expression patterns across various tissues, with notably higher levels in cotyledon, unifoliate, and trifoliate tissues (Figure S1d, Supporting Information). Furthermore, under long‐day conditions, these genes undergo periodic changes in their expression (Figure S1e, Supporting Information). Additionally, both GmMTAa and GmMTAb are nucleocytoplasmic proteins, localizing to both the nucleus and cytoplasm (Figure S1f, Supporting Information).

**Figure 1:**
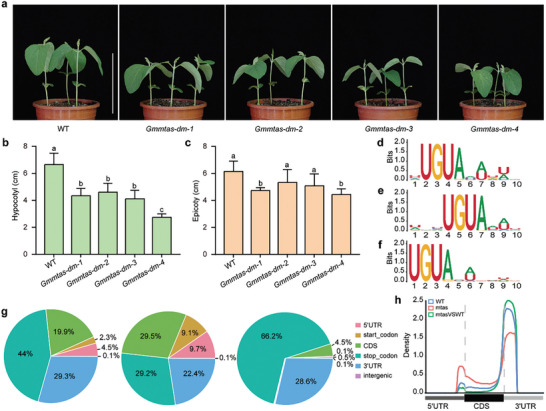
Disruption of *GmMTAs* results in m^6^A hypomethylation of mRNAs and growth inhibition of hypocotyls. a, Representative images of *GmMTAs* double plants grown in LD conditions. Scale bar, 10 cm. b,c, Statistical analysis of the hypocotyl (b) and epicotyl (c) of the indicated lines as in (a). Data are shown as means ± SD (n ≥ 4) with Ordinary one‐way ANOVA, followed by a Tukey multiple comparisons posttest (*P* < 0.05). d‐f, The MEME‐identified motifs for those m^6^A peaks in WT (d), *Gmmtas‐dm‐4* (e) and hypomethylated m^6^A peaks in *Gmmtas‐dm‐4* (f). g) Pie chart presenting the fractions of m^6^A peaks identified in WT, *Gmmtas‐dm‐4* and the hypomethylated m^6^A peaks in *Gmmtas‐dm‐4* among transcript segments. h, Metagene profile presenting the distributions of m^6^A peaks identified in WT, *Gmmtas‐dm‐4* and the hypomethylated m^6^A peaks in *Gmmtas‐dm‐4* across the indicated mRNA segments.

To better understand the functional role of *GmMTAs* in soybean development, we utilized CRISPR/Cas9 technology to generate single mutations in *Gmmtaa* and *Gmmtab*, as well as double mutations in these *Gmmtas* genes (Figures S2a,b, and S3, Supporting Information). Phenotypic analysis under long‐day, natural long‐day, and monochromatic light conditions revealed that all mutant variants exhibited shorter hypocotyl lengths, shorter epicotyl lengths, and reduced overall plant heights compared to wild‐type (WT) plants (**Figure** **1**a–c; Figures S2a–e,S4, and S5, Supporting Information). Notably, the double mutant displayed an enhanced phenotype, with the *Gmmtas*‐*dm*‐*4* mutant showing the most pronounced effects. Additionally, we generated lines overexpressing *GmMTAa* and *GmMTAb*, which specifically under long‐day conditions, exhibited increased hypocotyl lengths (Figure S6, Supporting Information).

To investigate the role of GmMTAs in m6A methylation in soybean, we conducted methylated RNA immunoprecipitation sequencing (MeRIP‐Seq) on both WT and *Gmmtas‐dm‐4* plants at ZT 4 under long‐day conditions. We first mapped the clean reads to the transcriptome via HISAT2 (http://daehwankimlab.github.io/hisat2), and 88.61–90.54% of the clean reads were successfully mapped to the soybean reference genome (*Glycine max Wm8*2*.a2.v1*) (Figure S7a, Supporting Information). The sequencing results yielded several noteworthy observations. First, we consistently detected the presence of the methylation motif UKUGUAW (K = U or G; W = U or A) in both the WT and *Gmmtas‐dm‐4* plants and hypomethylated m6A peaks in the *Gmmtas‐dm‐4* plants (Figure 1d–f). However, significant differences were observed in the number of methylated genes and the distribution of methylation peaks in these genes. After comparing the m6A peaks between the WT and *Gmmtas‐dm‐4* plants, we identified a total of 13,330 confidently hypomethylated m6A peaks, corresponding to 13 010 genes in the *Gmmtas‐dm‐4* plants (Figure S7b,c, Supporting Information). Notably, these hypomethylated m6A peaks were more highly enriched near stop codons and within the 3'UTR (Figure 1g,h). Further analysis was conducted to elucidate the functional implications of these hypomethylated m6A modifications. Gene Ontology (GO) enrichment analysis revealed that the genes associated with hypomethylated m6A modifications were significantly involved in processes related to “protein transport” and “cytoplasmic translation” (Figure S7d, Supporting Information). Additionally, KEGG pathway analysis demonstrated that these proteins were enriched in pathways such as “ubiquitin‐mediated proteolysis” and “peroxisome” (Figure S7e, Supporting Information). Taken together, our results demonstrate that GmMTAs also act as m6A writers in soybean.

### 2.2 Disruption of GmMTAs leads to Hypomethylation of *GmCRY1s* mRNA

To further explore the underlying mechanism through which *GmMTAs* regulate hypocotyl elongation, we examined genes located within the hypomethylated m6A peaks and pinpointed a set of crucial genes involved in this process, including the blue light signaling pathway genes *GmCRY1s*, *GmSPAs*, and *GmCOP1s* (Table S1, Supporting Information). It is noteworthy that in *Arabidopsis*, *CRY1* has been established as a key player in mediating the inhibitory effect of blue light on hypocotyl elongation.^[^ ^36^, ^37^, ^38^ ^]^ Consistent with these findings, previous research in soybean has shown that overexpression of *GmCRY1b* results in a dwarf phenotype reminiscent of that seen in *Gmmta* mutants.^[^ ^8^ ^]^

**Figure 2:**
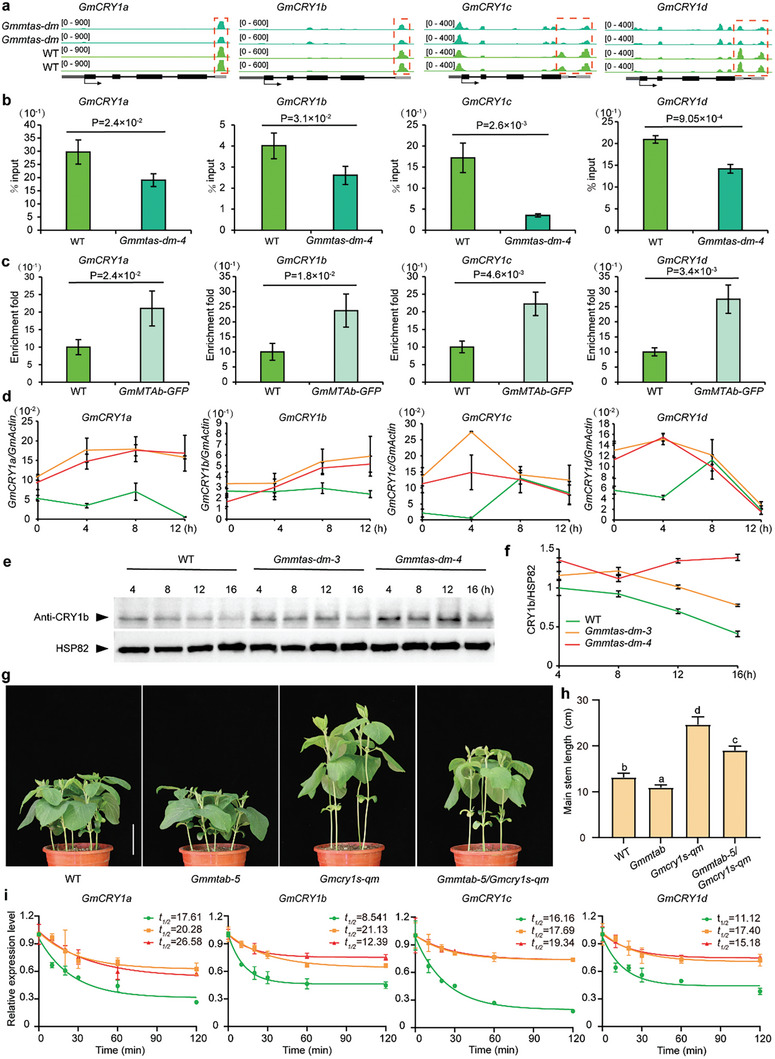
MTA‐dependent m^6^A methylation suppresses mRNA and protein expression of *GmCRY1s*. a) Genomic visualization of m^6^A density maps of *GmCRY1s*. Gene structures are displayed below the m^6^A density map with the 5’UTR (dark grey box), exon (black box), intron (black line) and 3’UTR (light grey) shown. Arrows indicate the direction of transcription. The red dotted lines indicate the location of the hypomethylation of m^6^A peaks. b) m^6^A abundance of individual sites of *GmCRY1s* were analyzed by m^6^A‐IP qPCR under long day conditions for 7 days. The epicotyl and single leaves were collected at ZT 4. Each sample was analyzed in triplicate. Data are shown as means ± SD (n = 3) for three biological replicates with significant difference by Student's t‐tests. c) RNA immunoprecipitation assay reveals the direct binding of GmMTAb‐GFP to the transcripts of *GmCRY1s*. Seven days old wild type (Tianlong 1) and *GmMTAs‐GFP* seedlings grown under long days were harvested at ZT 4. Input as a negative control. Data are shown as means ± SD (n = 3) for three biological replicates. d) Analysis of the *GmMTAs* effect on the expression of *GmCRY1s*. The first fully expanded unifoliolate leaves were collected at ZT 0, ZT 4, ZT 8 and ZT 12. Each sample was analyzed in triplicate. e) Comparison of GmCRY1b protein levels in *Gmmtas‐dm* mutants under long day conditions by immunoblot using GmCRY1b antibody. The first young trifoliolate leaves were collected at ZT 4, ZT 8, ZT 12 and ZT 16. Each sample was analyzed in triplicate. HSP82 was used as the loading control. f) Quantitative assay of GmCRY1b protein levels relative to HSP82 in samples as in (e). Values are mean ± SD (n = 3 biological replicates). The levels of GmCRY1b protein were calculated by normalization of their signal relative to HSP82 signals and are presented as relative expression units (REU = signal intensity of GmCRY1b/signal intensity of HSP82). g) *Gmmtab‐5*/*Gmcry1s‐qm* mutants plant height lower than *Gmcry1s‐qm*. Scale bar, 5 cm. h) Statistical analysis of the plant height of the indicated lines as in (g). Data are shown as means ± SD (n ≥ 4) with Ordinary one‐way ANOVA, followed by a Tukey multiple comparisons posttest (*P* < 0.05). i) qPCR assays showing mRNA lifetimes of *GmCRY1s* under continuous white light. Data are represented as means ± SD for 3 biological replicates × 2 technical replicates. The soybean *GmActin* gene *(Glyma.18G290800*) was used as an internal control. The quantitative PCR results using the formula: ΔCt = Ct (gene) – Ct (Actin), then gene expression level = 2^−^ΔCt.

To further elucidate the functional interplay between *GmMTAs* and *GmCRY1s*, we initially performed m6A‐IP qPCR on epicotyl and unifoliate samples collected at ZT 4 under long‐day conditions. The results revealed a notable decrease in m6A levels in the *Gmmtas‐dm‐4* mutants compared to the WT plants (Figure 2b), aligning with our m6A‐seq analysis findings (**Figure** **2**a). Building on this, we conducted RNA immunoprecipitation (RIP)‐qPCR using 7‐day‐old leaves from both WT and *Gmmtas*‐*dm*‐*4* plants, also harvested at ZT 4 under long‐day conditions. This analysis showed that the GmMTAb protein directly interacts with GmCRY1s transcripts (Figure 2c), collectively indicating that *GmCRY1s* are direct targets of GmMTAs. Furthermore, quantitative real‐time PCR analysis unveiled a significant upregulation of *GmCRY1s* transcription in *Gmmtas*‐*dm* mutants under long‐day conditions (Figure 2d). Western blotting analysis also demonstrated higher levels of the GmCRY1b protein in Gmmtas‐dm mutants compared to WT plants (Figure 2e,f), suggesting an inverse correlation between the expression of *GmMTAs* and the abundance of the GmCRY1b protein.

To explore the genetic relationship between *GmMTAs* and *GmCRY1s*, we crossed the *Gmmtab‐5* mutant with the *Gmcry1s‐qm* mutant. The resulting plants exhibited an intermediate plant height phenotype, and they were taller than *Gmmtab‐5* but shorter than *Gmcry1s*‐*qm* (Figure 2g,h). This indicates that the *Gmcry1s*‐*qm* mutant partially suppresses the dwarf plant height phenotype of the *Gmmtab‐5* mutant. Similarly, when we crossed *Gmmtab‐5* and *Gmmtas‐dm‐1* with *GmCRY1b*‐*YFP*, all the resulting plants displayed the shorter plant height phenotype of *GmCRY1b*‐*YFP* (Figure S8, Supporting Information). Therefore, we can conclude that *GmCRY1s* act downstream of *GmMTAs* in the regulation of plant height, but they are not the only genes involved in this regulation.

Considering that m6A modification can affect mRNA stability in humans^[^ ^13^, ^39^ ^]^ and *Arabidopsis* ^[^ ^20^, ^40^, ^41^, ^42^ ^]^ and that GmMTAs mediate m6A modification of *GmCRY1s* mRNA, we conducted transcription inhibition assays using cordycepin to assess the lifespan of *GmCRY1s* transcripts. The results indicated that *GmCRY1s* transcripts degrade more slowly in *Gmmtas‐dm* mutants than in WT plants. Overall, the loss of *GmMTA* function reduces m6A in *GmCRY1s* and increases the stabilization of *GmCRY1s* mRNA.

### 2.3 GmMTAs Promote *GmSPAs* Expression to Control Plant Height

**Figure 3:**
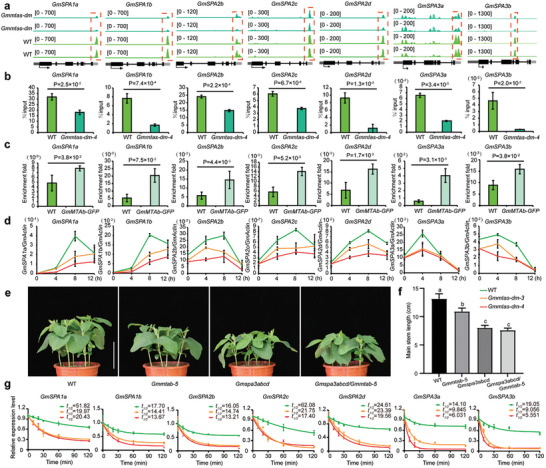
*GmMTAs*‐mediated m^6^A methylation modulates *GmSPAs* expression in plant height control. a) Genomic visualization of m^6^A density maps of *GmSPAs*. Gene structures are displayed below the m^6^A density map with the 5’UTR (dark grey box), exon (black box), intron (black line) and 3’UTR (light grey) shown. Arrows indicate the direction of transcription. The red dotted lines indicate the location of the hypomethylation of m^6^A peaks. b) m^6^A abundance of individual sites of *GmSPAs* were analyzed by m^6^A‐IP qPCR under long day conditions for 7 days. The epicotyl and single leaves were collected at ZT 4. Each sample was analyzed in triplicate. Data are shown as means ± SD (n = 3) for three biological replicates with significant difference by Student's t‐tests. c) RNA immunoprecipitation assay reveals the direct binding of GmMTAb‐GFP to the transcripts of *GmSPAs*. Seven days old wild type (Tianlong 1) and *GmMTAb‐GFP* seedlings grown under long days were harvested at ZT 4. Input as a negative control. Data are shown as means ± SD (n = 3) for three biological replicates. d) Analysis of the *GmMTAs* effect on the expression of *GmSPAs*. The first fully expanded unifoliolate leaves were collected at ZT 0, ZT 4, ZT 8 and ZT 12. Each sample was analyzed in triplicate. e) *Gmmtab‐5*/*Gmspa3abcd* mutants plant height were consistent with *Gmspa3abcd*. Scale bar, 5 cm. f) Statistical analysis of the plant height of the indicated lines as in (e). Data are shown as means ± SD (n ≥ 4) with Ordinary one‐way ANOVA, followed by a Tukey multiple comparisons posttest (*P* < 0.05). g) qPCR assays showing mRNA lifetimes of *GmSPAs* under continuous white light. Data are represented as means ± SD for 3 biological replicates × 2 technical replicates. The soybean *GmActin* gene *(Glyma.18G290800*) was used as an internal control. The quantitative PCR results using the formula: ΔCt = Ct (gene) – Ct (Actin), then gene expression level = 2^−^ΔCt.

Next, we turned our attention to investigating the role of *GmSPAs*. Previous studies on soybean indicated that *Gmspa3ab* (*phd*) exhibited a dwarf phenotype, similar to the phenotype observed in the *Gmmta* mutants.^[^ ^9^ ^]^ The soybean genome encodes 10 *GmSPA* genes, 7 of which exhibit persistent hypomethylated m6A peaks (**Figure** **3**a). We first validated the decrease in the m6A levels in *GmSPAs* from the *Gmmtas‐dm‐4* mutants through m6A‐IP qPCR analysis (Figure 3b), and the results were consistent with the m6A‐seq results. RNA immunoprecipitation assays also demonstrated the direct binding of GmMTAb to *GmSPAs* mRNA (Figure 3c), indicating that GmMTAs directly modulate m6A methylation in *GmSPAs* transcripts. Moreover, quantitative real‐time PCR revealed significant downregulation of *GmSPAs* expression in *Gmmtas‐dm* mutants compared to that in WT plants (Figure 3d).

To investigate the genetic relationship between *GmMTAs* and *GmSPAs*, we crossed the *Gmmtab‐5* mutant with the *Gmspa3abcd* mutant. The resulting phenotype was consistent with that of the *Gmspa3abcd* mutant (Figure 3e,f), indicating that *GmSPAs* act downstream of *GmMTAs* in the regulation of plant height.

Subsequently, we analyzed the stability of *GmSPAs* mRNA in both the WT and *Gmmtas‐dm* mutants. Notably, *GmSPAs* mRNA degraded faster in the *Gmmtas‐dm* mutant than in WT plants (Figure 3g), suggesting that GmMTAs affect the *GmSPAs* stability through m6A methylation, thereby regulating plant height.

### 2.4 GmMTAs Promote *GmCOP1s* Expression to Regulate Plant Height

**Figure 4:**
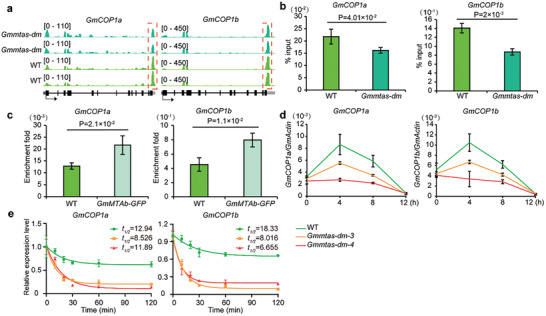
*GmMTAs*‐mediated m^6^A methylation modulates *GmCOP1s* expression in plant height control. a) Genomic visualization of m^6^A density maps of *GmCOP1a* and *GmCOP1b*. Gene structures are displayed below the m^6^A density map with the 5’UTR (dark grey box), exon (black box), intron (black line) and 3’UTR (light grey) shown. Arrows indicate the direction of transcription. The red dotted lines indicate the location of the hypomethylation of m^6^A peaks. b) m^6^A abundance of individual sites of *GmCOP1s* were analyzed by m^6^A‐IP qPCR under long day conditions for 7 days. The epicotyl and single leaves were collected at ZT 4. Each sample was analyzed in triplicate. Data are shown as means ± SD (n = 3) for three biological replicates with significant difference by Student's t‐tests. c) RNA immunoprecipitation assay reveals the direct binding of GmMTAb‐GFP to the transcripts of *GmCOP1s*. Seven days old wild type (Tianlong 1) and *GmMTAb‐GFP* seedlings grown under long days were harvested at ZT 4. Input as a negative control. Data are shown as means ± SD (n = 3) for three biological replicates. d) Analysis of the *GmMTAs* effect on the expression of *GmCOP1s*. The first fully expanded unifoliolate leaves were collected at ZT 0, ZT 4, ZT 8 and ZT 12. Each sample was analyzed in triplicate. e, qPCR assays showing mRNA lifetimes of *GmCOP1s* under continuous white light. Data are represented as means ± SD for 3 biological replicates × 2 technical replicates. The soybean *GmActin* gene *(Glyma.18G290800*) was used as an internal control. The quantitative PCR results using the formula: ΔCt = Ct (gene) – Ct (Actin), then gene expression level = 2^−^ΔCt.

*CONSTITUTIVE PHOTOMORPHOGENIC 1* (*COP1*) is a key photomorphogenic inhibitor that enhances soybean performance under dense planting conditions.^[^ ^10^ ^]^ We found that the m6A modification levels in the 3'UTRs of *GmCOP1a* and *GmCOP1b* were lower in the *Gmmtas‐dm‐4* mutant than in WT plants (**Figure** **4**a,b). RNA immunoprecipitation assays revealed the direct binding of GmMTAb to *GmCOP1s* (Figure 4c), indicating that GmMTAs directly modulate m6A methylation in *GmCOP1s* transcripts. Furthermore, quantitative real‐time PCR showed decreased expression of *GmCOP1s* (Figure 4d). Interestingly, mutations in *GmMTAs* genes led to a shortened half‐life of *GmCOP1s* mRNA or accelerated its degradation in *Gmmtas‐dm* seedlings when compared to that in WT seedlings (Figure 4e). Therefore, GmMTAs also influence the m6A modification and transcription of *GmCOP1s*, subsequently regulating the height of soybean plants.

### 2.5 GmMTAs Suppress *GmSTFs* mRNA and Protein Expression

Recent investigations in soybean have demonstrated that *GmCRY1s*, *GmSPAs*, and *GmCOP1s* play significant roles in regulating plant height through the involvement of *GmSTFs*.^[^ ^8^, ^9^, ^10^ ^]^ Based on these findings, we propose the hypothesis that GmMTAs may regulate soybean plant height by modulating the *GmCRY1s*/*GmSPAs*/*GmCOP1s*‐*GmSTFs* signalling pathway.

To test this hypothesis, we initially examined the impact of GmCRY1s on the protein interaction between GmSPAs and GmCOPs under blue light conditions. The Yeast three‐hybrid assays revealed that, when GmCRY1b was absent, the presence or absence of blue light did not affect the GmCOP1a/b‐GmSPA3a interaction. However, when GmCRY1b was present and yeast cells were exposed to blue light, the interaction between GmCOP1a/b and GmSPA3a was significantly suppressed (Figure S9, Supporting Information).

**Figure 5:**
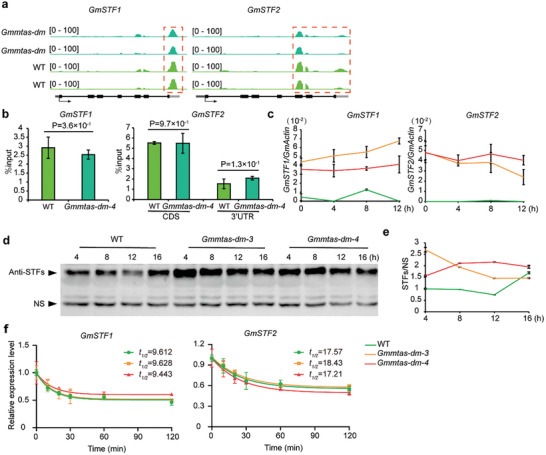
*GmMTAs* inhibit expression of the *GmSTFs* genes involved in the regulation of plant height. a) Genomic visualization of m^6^A density maps of *GmSTFs*. Gene structures are displayed below the m^6^A density map with the 5’UTR (dark grey box), exon (black box), intron (black line) and 3’UTR (light grey) shown. Arrows indicate the direction of transcription. The red dotted lines indicate the location of the m^6^A peaks. b) m^6^A abundance of individual sites of *GmSTFs* were analyzed by m^6^A‐IP qPCR under long day conditions for 7 days. The epicotyl and single leaves were collected at ZT 4. Each sample was analyzed in triplicate. Data are shown as means ± SD (n = 3) for three biological replicates with significant difference by Student's t‐tests. c) Analysis of the *GmMTAs* effect on the expression of *GmSTFs*. The first fully expanded unifoliolate leaves were collected at ZT 0, ZT 4, ZT 8 and ZT 12. Each sample was analyzed in triplicate. d) Comparison of GmSTFs protein levels under long day condition by immunoblot using GmSTFs antibody. The first young trifoliolate leaves were collected at ZT 4, ZT 8, ZT 12 and ZT 16. Each sample was analyzed in triplicate. A non‐specific band (NS) was used as the loading control. e) Quantitative assay of GmSTFs protein levels relative to NS in samples as in (d). Values are mean ± SD (n = 3 biological replicates). The levels of *GmSTFs* proteins were calculated by normalization of their signals relative to NS signals and are presented as relative expression units (REU = signal intensity of *GmSTFs*/signal intensity of NS). f) qPCR assays showing mRNA lifetimes of *GmSTFs* under continuous white light. Data are represented as means ± SD for 3 biological replicates × 2 technical replicates. The soybean *GmActin* gene *(Glyma.18G290800*) was used as an internal control. The quantitative PCR results using the formula: ΔCt = Ct (gene) – Ct (Actin), then gene expression level = 2^−^ΔCt.

We next examined the m6A levels in *GmSTF1* and *GmSTF2* in both WT and *Gmmtas*‐*dm*‐4 mutant plants. Both the m6A‐IP qPCR and m6A‐seq results revealed that compared to those WT plants, the methylation levels of *GmSTFs* were not different in the *Gmmtas‐dm‐4* mutant (**Figure** **5**a,b). This indicates that GmMTAs do not directly regulate the m6A modification of *GmSTFs*. We further evaluated the transcription and protein levels of *GmSTF1* and *GmSTF2* using quantitative real‐time PCR and western blotting analysis, respectively. Consistent with the observed phenotypes in *Gmmtas‐dm* mutants, our results showed that the expression and protein levels of *GmSTFs* were greater in *Gmmtas‐dm* mutants than in WT plants under long‐day conditions (Figure 5c–e). Moreover, the stability of *GmSTFs* mRNA remained largely unchanged in *Gmmtas‐dm* seedlings compared to WT seedlings (Figure 5f), indicating that GmMTAs indirectly regulate the RNA and protein levels of *GmSTFs*.

Additionally, we examined the expression levels of *GmSTFs* in *Gmspas*, *GmCRY1b*‐*YFP*, and *Gmcry1s‐qm* plants after exposure to continuous white light. Consistent with our previously published results,^[^ ^8^, ^9^, ^10^ ^]^ *GmSTF1* and *GmSTF2* were increased in *Gmspas* and *GmCRY1b*‐*YFP* plants and decreased in *Gmcry1s*‐*qm* plants compared to WT plants (Figure S10, Supporting Information). In addition, our previous research found that the changes in GmSTF1 and GmSTF2 protein were consistent with their changes in expression in *Gmspas*, *GmCRY1b*‐*YFP*, *Gmcop1s*, and *Gmcry1s‐qm* plants.^[^ ^8^, ^9^, ^10^ ^]^ These findings indicate that *GmMTAs* mediate m6A methylation to regulate the expression of *GmCRY1s*, *GmSPAs, and GmCOP1s*, which indirectly controls the abundance of *GmSTFs*. Thus, our results provide further evidence supporting the role of *GmMTAs* in regulating soybean plant height through the GmCRY1s/GmSPAs/GmCOP1s‐GmSTFs pathway.

### 2.6 *GmMTAs* Regulate LBL‐Induced ESE

**Figure 6:**
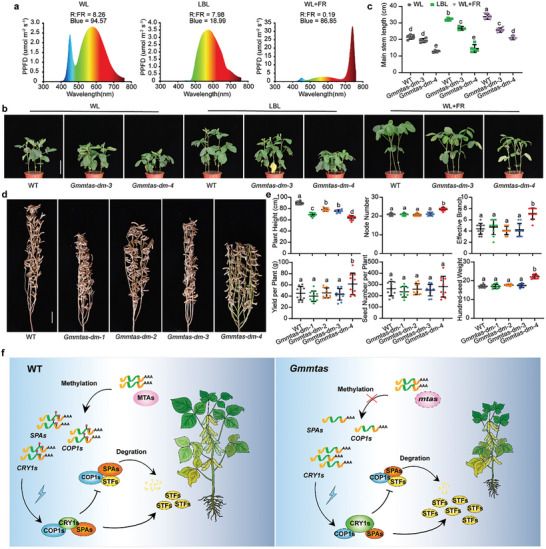
*GmMTAs* regulates shade avoidance induced by low blue light. a) Light spectral compositions of white light (WL), low blue light (LBL), WL plus far‐red light (WL+FR). Low R:FR was achieved by supplementing far‐red light to WL; LBL was achieved by filtering WL through two layers of yellow filters. The light quality and intensity of WL, LBL and WL+LBL were all 500 µmol·m^−2^·s^−1^. b) Representative images of the indicated lines grown under WL, LBL and WL+FR conditions. 10 days seedlings were treated with the indicated light regimes in (a) under long day conditions for 10 days. Scale bar, 10 cm. c) Statistical analysis of the plant height of each line in (b). Data are shown as means ± SD (n ≥ 4). The letters indicate significant differences with Ordinary one‐way ANOVA, followed by a Tukey multiple comparisons posttest (*P* < 0.05). d) Representative images of *Gmmtas* double mutants grown in Yihai, Beijing in the summer of 2023, China (40.1°N, 116.7°E). Scale bar, 10 cm. e) Statistical analysis of the agronomic traits of the indicated lines as in (d). Data are shown as means ± SD (n = 10) with significant difference by Ordinary one‐way ANOVA, followed by a Tukey multiple comparisons posttest (*P* < 0.05). f) Hypothetical model demonstrating the mechanism of *GmMTAs*‐mediated blue light signal regulating plant height and shade avoidance in soybean. In *Gmmtas* mutants, the methylation modification levels of *GmCRY1s*, *GmSPAs* and *GmCOP1s* were reduced, resulting in increased transcription levels of *GmCRY1s* and decreased transcription levels of *GmSPAs* and *GmCOP1s*. GmCRY1s can competitively bind GmSPAs, making GmCOP1s/GmSPAs complexes unable to degrade GmSTFs, resulting in lower plant height.

Previous studies have established that GmCRY1s, GmSPAs, and GmCOP1s regulate the shade avoidance response of soybean plants under low blue light (LBL) conditions, mediated by GmSTFs.^[^ ^8^, ^9^, ^10^ ^]^ To elucidate the role of GmMTAs in this process, we exposed 10‐day‐old WT and *Gmmtas‐dm* mutant seedlings to different light conditions—white light (WL), LBL, and WL combined with far‐red light (WL+FR) under long‐day conditions for 10 days (**Figure** **6**a; Figure S11a, Supporting Information). Our findings revealed that both the WT and *Gmmtas‐dm* mutant plants exhibited increased height after exposure to LBL and WL+FR. However, the *Gm*mta*s‐dm* mutants consistently exhibited shorter height than the WT plants across all lighting conditions (Figure 6b,c; Figure S11b,c, Supporting Information). Notably, the *Gmmtas‐dm‐4* mutant plants exhibited comparable heights under the WL and LBL treatments (Figure 6b,c), suggesting a significant suppression of the LBL‐induced shade‐avoidance response. Additionally, we observed similar inhibitory effects on the shade‐avoidance response in *Gmspa3ab* and *Gmspa3abcd* mutants, as well as in plants overexpressing *GmCRY1b* and *GmSTF1* (Figure S12, Supporting Information). These results collectively implicate GmMTAs in the regulation of the shade avoidance response, likely through the GmCRY1s/GmSPAs/GmCOP1s‐GmSTFs pathway.

To investigate the performance of *Gmmtas* mutants under field conditions, we conducted field trials in Yihai, Beijing for two consecutive years (2022 and 2023). Aside from the reduction in plant height, the main stem node number, effective branching, yield per plant, number of seeds, and 100‐seed weight of the *Gmmtas* mutants remained unchanged (Figure 6d,e; Figures S13,S14, Supporting Information). Interestingly, compared with the WT plants, the *Gmmtas*‐*dm*‐*4* mutant plants exhibited several favorable agronomic traits, including a greater number of main stem nodes, more branches, greater yield per plant, and greater 100‐seed weight (Figure 6d,e). Furthermore, we also conducted field trials with *Gmmtas* double mutants in Shunyi and Changchun in 2023. As expected, in addition to the reduction in plant height, the main stem node number, effective branching, yield per plant, and number of seeds of the *Gmmtas* mutants remained unchanged (Figure S15, Supporting Information). Notably, the 100‐seed weight increased in Changchun, possibly due to a longer fertility period (Figure S15c,d, Supporting Information). These results demonstrate the potential of targeted mutagenesis of the *GmMTA* genes to result in increased yields under high‐density planting conditions.

## 3 Discussion

In this study, we conducted a comprehensive investigation into the molecular mechanisms that underlie soybean's response to low blue light (LBL) conditions. LBL triggers the SAS, resulting in a series of physiological changes, notably abnormal stem elongation and yield reduction.^[^ ^8^ ^]^ Our findings reinforce the established roles of conserved signaling molecules, including GmCRY1s^8^, GmSPAs^9^, and GmCOP1s^10^, in modulating plant height and mediating SAS across species. Importantly, we uncover a novel aspect of epigenetic regulation—m6A methylation—within this signaling cascade, and demonstrate that the key components of the GmCRY1s/GmSPAs/GmCOP1s‐STFs axis are regulated by m6A methylation (Figure 6f), highlighting its potential significance in fine‐tuning plant growth and development under varying light environments.

The m6A modification profoundly influences mRNA stability, delicately balancing persistence and turnover within cells. Paradoxically, this epigenetic mark can both reinforce mRNA stability, thereby enhancing its lifespan and translational potential and facilitate rapid degradation in response to cellular needs.^[^ ^13^, ^20^, ^39^, ^40^, ^41^, ^42^ ^]^ As a central recognition hub, m6A orchestrates mRNA turnover by engaging with m6A RNA‐binding proteins (RBPs), or ‘readers’, such as YTHDF2, which directs the HRSP12–RNase P–MRP^[^ ^43^ ^]^ and CCR4‐NOT^[^ ^44^ ^]^ complexes to degrade m6A‐tagged mRNAs. In contrast, m6A may impede degradation under specific conditions by interacting with readers like PRRC2A,^[^ ^45^ ^]^ illustrating its versatility in fine‐tuning mRNA stability. This intricate regulatory mechanism ensures precise mRNA level adjustments, empowering cells to swiftly adapt to environmental fluctuations while intricately intertwining with other mRNA modifications, species, and decay pathways. Transcription dynamics serve as a vital buffer, modulating RNA degradation rates.

Our investigation of *Gmmtas‐dm* mutants unveils a selective upregulation of *GmCRY1s* and downregulation of *GmSPAs* and *GmCOP1s* expression following reduced m6A methylation (Figures 2, 3, 4), with alterations in mRNA stability playing a pivotal role in this process. In addition, our unpublished data shows that GmECT2, a YTHDF2‐like protein, is a regulatory factor for *GmCRY1s* expression. In ECT2 mutants, both *GmCRY1s* mRNA and protein levels are upregulated, whereas *GmSPAs* and *GmCOP1s* remain largely unchanged. This observation further supports the notion that reader proteins have selective recognition of m6A markers and underscores the urgency for deeper exploration into the molecular mechanisms governing m6A methylation recognition and its functional implications across diverse plant genes.

Our investigation into soybean GmMTAs has highlighted the complex interaction between evolutionary conservation and functional divergence in mRNA m6A methylation. Specifically, the *Gmmtas*‐*dm*‐*4* double knockout mutant, which lacks both *GmMTA* genes, exhibits a non‐lethal phenotype, contrasting sharply with the embryo‐lethal phenotype observed in the *Arabidopsis mta* mutant.^[^ ^22^ ^]^ Concurrently, our results confirm the involvement of soybean GmMTAs in the CRY signal transduction pathway,^[^ ^15^, ^46^ ^]^ albeit soybean GmMTAs may mediate light responses by different mechanisms. Strikingly, the Gmmtas‐dm‐4 knockout mutants display enhanced agronomic traits compared to wild‐type plants, including increased stem node count, branch number, yield per plant, and heavier 100‐seed weight (Figure 6d,e). This divergence highlights the potential for functional variation between model organisms and crops, offering crucial insights for agricultural enhancement.

Re‐examining model organism research in the context of crops is crucial, as it reveals opportunities for translational advancements that extend beyond basic science. Our comprehensive analysis of m6A methylation in soybean's response to LBL conditions sheds light on this epigenetic mark's evolutionary conservation and functional divergence across species. These findings deepen our molecular understanding of plant growth and development and pave the way for genetic improvements in crops, with the overarching goal of enhancing global food security.

## 4 Experimental Section

### Plant Materials and Growth Conditions

All wild type (WT), overexpressed and mutant plants used in this study were Tianlong 1 accessions. The Williams 82 (W82) cultivar was used for tissue‐specific and rhythmic expression assays, which were all planted under long‐day conditions for 3 weeks, the tissues of root, hypocotyl, epicotyl, stem, cotyledon, unifoliate leaves, trifoliolate leaves, flowering, and stem tip were collected for tissue‐specific expression analysis of *GmMTAa* and *GmMTAb*. The trifoliolate leaves were collected for the rhythmic expression of *GmMTAa* and *GmMTAb*. For phenotypic observation, all transgenic plants were planted under long‐day conditions (16 h light/8 h dark, 27 °C) in the growth chamber and under natural long‐day conditions in Beijing (40.1° N,116.7° E); Shunyi, Beijing (40.2°N, 116.6°E) and Changchun, Jilin (43.8°N, 125.4°E). Above transgenic plants were grown with a plant spacing of 10 cm and a row spacing of 50 cm. For m6A methylation analysis, WT and mutant plants were planted under long‐day conditions for 7 days, and the epicotyl and unifoliate leaves were collected at ZT 4, with more than 8 plants as a replicate.

### Plasmid Construction and Acquisition of Transgenic Plants

To construct the overexpression vectors of *GmMTAa* and *GmMTAb*, primers were designed according to the reference genome *Wm82.a2.v1*, and the full‐length CDS coding sequences of *GmMTAa* and *GmMTAb* were amplified using the cDNA of Williams 82 of young leaves. The full‐length CDS of *GmMTAa* (2286 bp) and *GmMTAb* (2283 bp) were inserted into the overexpression vectors of the *PTF101*‐*GFP* and *0641*‐*Flag* using the In‐fusion system. To generate the CRISPR/Cas9 engineered *Gmmtaas* mutants, guide‐RNAs were designed using the CRISPR‐P website (http://crispr.dbcls.jp/). Sequences containing target sites were inserted into the CRISPR/Cas9 vector using In‐fusion and T4 systems. The CRISPR/Cas9 vector was modified and preserved in our laboratory.^[^ ^47^ ^]^ All knockout vectors were transferred into the *Agrobacterium tumefaciens* strain K599, which was used to verify the working efficiency using soybean hair root induction technique according to a previously published method.^[^ ^48^ ^]^ The overexpression and CRISPR/ Cas9 vectors were transferred into *Agrobacterium tumefaciens* strain *EH105* via electroporation and then infected WT (Tianlong 1) using the cotyledon‐node method.^[^ ^49^ ^]^ Primers for the detection of transgenic plants are shown in Table S2 (Supporting Information).

### Accession Numbers

Accession numbers for all soybean genes reported in this study can be found in the Phytozome database (https://phytozome.jgi.doe.gov/), see Table S3 (Supporting Information) for details.

### RNA Extraction, Reverse Transcription, and Quantitative Real‐Time PCR

Each sample contained three biological replicates and three plants were mixed into one tube. Total RNA was extracted from all the samples using TRIzol reagent (Invitrogen). For reverse transcription, cDNA was synthesized using a reverse transcription kit (TRAN), and the total RNA amount should not exceed 5 ug. The quantitative real‐time PCR was performed by a two‐step method and the calculation formula was 2^−ΔCt^, ΔCt = CT(gene)‐CT (*GmActin*).

### Analysis of Subcellular Localization

To analyze the subcellular localization of *GmMTAa* and *GmMTAb*, the CDS of *GmMTAa* and *GmMTAb* were inserted into the PA7‐YFP vector at the *Xma*I and *BamH*I site using an In‐fusion system. The PA7‐YFP empty vector was used as a control. Prepared *Arabidopsis* protoplasts by incubating young leaves in a 10 mL enzyme solution (0.15 g Cellulase R10, 0.04 g Macerozyme R10, 0.728 g D‐Mannitol, 20 mM MES pH = 5.7, 20 mM KCl, 10 mM CaCl_2_) for 3–4 h in darkness. Filtered protoplasts through a 200 mesh screen, then centrifuged at 100 g for 2 min at 4 °C. Washed protoplasts twice with 10 mL pre‐cooled W5 buffer (154 mM NaCl, 5 mM KCl, 2 mM MES pH = 5.7, 125 mM CaCl_2_), followed by a 30 min incubation on ice. Collected and resuspended protoplasts in MMg buffer (0.728 g D‐Mannitol, 15 mM MgCl_2_, 4 mM MES pH = 5.7). Mixed 10 µL plasmids (1µg µL^−1^) with 100 µL protoplasts and 110 µL PEG (4 g PEG 4000, 0.364 g D‐Mannitol, 0.1 M CaCl_2_) at room temperature for 5–8 min in darkness. Washed protoplasts once with 440 µL W5 buffer, then twice with 500 µL W5 buffer, and finally resuspended in 1 mL W5 buffer in darkness. The subcellular localization images were captured under a Zeiss LSM980 confocal laser scanning microscope.

### Light Regimes

For the LBL treatment, white light (WL) was filtered through two layers of yellow filter film (no. 101, Lee Filters, CA, United States) as described previously.^[^ ^8^ ^]^ For the WL+ FR treatment, the far‐red LED panels were used to reduce the R/FR ratio. The light quality and intensity of WL, LBL, and WL+LBL were all 500 µmol·m^−2^·s^−1^, measured by HiPoint HR‐350 Spectrometer.

### Immunoblot Assay

The anti‐GmSTFs and anti‐GmCRY1b polyclonal antibodies used in this study were consistent with previous studies.^[^ ^8^ ^]^ The antibody anti‐GFP antibody (598) was obtained from MBL. The fresh leaves were ground into a powder with liquid nitrogen, and mixed with 4 × SDS‐PAGE loading buffer (200 mM Tris‐HCl pH = 6.8, 8% SDS, 40% glycerol, 32 mM DTT, and 0.008% Bromophenol blue) for 10 min at 98 °C and centrifuged at 4° C for 10 min. Then, 3–5 µL supernatants were added into 10％ SDS‐ SDS‐PAGE followed by 90 v, 40 min, and 120 v, 80 min. Next, the wet transfer method was used to PVDF membranes and blotted with corresponding antibodies.

### m6A‐Seq (MeRIP‐seq) and Data Analysis

Total RNA was isolated and purified using TRIzol reagent (Invitrogen, Carlsbad, CA, USA). Dynabeads Oligo (dT) beads (No. 25–61005, Thermo Fisher, USA) were used to specifically capture the mRNA containing PolyA (polyadenosine) through two rounds of purification. The poly(A) mRNA was fragmented using the NEBNext® Magnesium RNA Fragmentation Module (E6150S, USA) at 86 °C for 7 min. Then the RNA fragments were incubated with m^6^A‐specific antibody (No. 202003, Synaptic Systems, Germany) in IP buffer (50 mM Tris‐HCl, 750 mM NaCl, and 0.5% Igepal CA‐630) supplemented with RNasin Plus RNase inhibitor (Promega) at 4 °C for 2 h with gentle rotation. Beads were then washed two times with 1×IP buffer and the RNA was eluted with Phenol and Chloroform. Then dissolved in RNase‐free H_2_O. Input poly(A)+ RNA and immunoprecipitated poly(A)+ RNA were used to construct libraries with an NEBNext Ultra II RNA Library Prep Kit. Finally, Sequencing was performed on illumina Novaseq 6000 instruments in pair‐end mode with 150 bp per read.

For m^6^A profiling, fastp software (https://github.com/OpenGene/fastp) was used to remove the reads that contained adaptor contamination, low‐quality bases, and undetermined bases with default parameters. HISAT2 (http://daehwankimlab.github.io/hisat2) was used to map reads to the reference genome (*Glycine max Wm82.a2.v1*). Mapped reads of IP and input libraries were provided for R package exomePeak (https://bioconductor.org/packages/exomePeak), which identifies m^6^A peaks with tdf or bigwig format that can be adapted for visualization on the IGV software (http://www.igv.org). MEME (http://memesuite.org) and HOMER (http://homer.ucsd.edu/homer/motif) were used for known motif finding followed by localization of the motif for peak summit. Called peaks were annotated by intersection with gene architecture using the R package ChIPseeker (https://bioconductor.org/packages/ChIPseeker).

### m^6^A‐IP‐qPCR Assays

M^6^A‐IP‐qPCR assays, were performed using the magna RIP kit (Millipore,17‐700) according to the previous description with only slight changes.^[^ ^21^ ^]^ Briefly, 50 µL beads were used and then immunoprecipitated with 5 µg anti‐m^6^A antibody (Synaptic Systems, 202003) at room temperature for 30 min–1 h. 60 µg total RNA per sample was used and then was fragmented with 0.272 g ZnCl_2_ (0.1M) at 94 °C for 100 s and then incubated with beads for at least 3 h or overnight. 30 µL of fragmented mRNAs was taken as the input control. After RNA was isolated and dissolved in 20 µL DEPC water. Reverse transcription was performed with random primers. The primers used for qPCR are listed in Table S4 (Supporting Information).

### RNA Immunoprecipitation (RIP)‐qPCR

RNA immunoprecipitation was performed as previously published^[^ ^50^ ^]^ with minor modifications. 2 g of 7‐day‐old seedlings of WT and *GmMTAb‐GFP* were collected and fixed with 1% formaldehyde under vacuum for 12 min. The fixed tissues were homogenized and lysed with cell lysis buffer (50 mM Tris‐HCl pH = 8.0, 10 mM EDTA pH = 8.0, 1% SDS, 1 mM DTT, 0.1 mM PMSF, a slice of Protease Inhibitor Cocktail (Roche), 160 U/µl RNase inhibitor). The protein extract was subjected to immunoprecipitation with anti‐GFP Agarose beads (gta‐10) at 4 °C for 4 h, followed by three times washing with washing buffer (150 mM NaCl, 20 mM Tris‐HCl pH = 8.0, 2 mM EDTA pH = 8.0, 0.1% SDS, 1% Triton X‐100, 0.1 mM PMSF, 80 U/µl RNase inhibitor). The beads were subsequently treated with 20 µg Proteinase K (Solarbio) at 65 °C for 1 h. The input and immunoprecipitated RNA were isolated using Phenol/chloroform/isoamyl alcohol, and then reversely transcribed into cDNA using a reverse transcription kit (TRAN). Relative enrichment of each gene was determined by qPCR and normalized to input. The primers used for qPCR are listed in Table S4 (Supporting Information).

### RNA Stability Analysis

An mRNA stability measurement assay in vivo was performed as previously described with minor modification15. Briefly, WT, *Gmmtas‐dm‐3*, and *Gmmtas‐dm‐4* seedlings were grown under continuous white light for 8 days, and then unifoliate leaves were incubated in vacuum buffer (15 mM sucrose, 1 mM KCl, 1 mM PIPES pH = 6.25 and 1 mM sodium citrate) for 15 min and added 1 mM cordycepin (HY‐N0262‐50mg). The samples were vacuumed for 2 min and these samples were referred to as 0 h samples. The remaining seedlings were vacuumed again for 2 min, and collected at 10, 20, 30, 60, and 120 min. The total RNA was extracted using FastPure Universal Plant Total RNA Isolation Kit (RC411‐01, Vazyme). cDNA was synthesized using a TransScript one‐step gDNA removal and cDNA synthesis supermix (AT311‐03, TRAN). The quantitative real‐time PCR was performed by a two‐step method using ChamQ universal SYBR qPCR master mix (Q711‐03, Vazyme), and the calculation formula was 2‐ΔCt,ΔCt = CT(gene)‐CT (GmActin). GmActin was used as the internal control. The T0 point of each sample was set as 1 to calculate the relative expression. The resulting mRNA decay curve was fitted with a one‐phase exponential decay model using GraphPad Prism 9 to estimate the half‐life (*t*_1/2_). The primers used for qPCR are listed in Table S4 (Supporting Information).

### Yeast Three‐Hybrid Assay

The CDS of *GmSPA3a* was fused with the bait vector pBridge to construct the *pBridge‐GmSPA3a* vector. The CDS of *GmCRY1b* was inserted into the *pBridge‐GmSPA3a* vector to generate the *pBridge‐GmSPA3a‐GmCRY1b* vector. The CDS of *GmCOP1a* and *GmCOP1b* were fused with the prey vector pGADT7 to make the *pGADT7‐GmCOP1a/GmCOP1b* vectors. The yeast strain AH109 was transformed with the indicated vector combinations. The plasmids were transformed into the yeast strain AH109 (Clontech). The multiple colonies were selected and cultured in 4 mL SD medium (−Leu/‐Met/−Trp/+Asp) at 30 °C, 220 rpm. Then 2 mL yeast culture was transferred into 8 mL YPDA medium until OD600 = 0.7‐1.0 under dark and continuous blue light (30 µmol·m^−2^·s^−1^), respectively, followed by β‐galactosidase assays.

## Conflict of Interest

The authors declare no conflict of interest.

## Author Contributions

L.Z. and J.L. contributed equally to this work. T.Z. conceived and designed the project. L.Z., J.C., Y.Z., C.Q., X.L., Z.L., and J.L. performed the experiments. L.Z. and T.Z. conducted the bioinformatic analyses. T.Z. and L.Z. wrote the manuscript. B.L. and H.L. revised the manuscript. All authors have read and agreed to the published version of the manuscript.

## Supporting information



Supporting Information

Supplemental Table 1

Supplemental Table 2

Supplemental Table 3

Supplemental Table 4

## Data Availability

The data that support the findings of this study are available in the supplementary material of this article.
