# Supplementary material for: Regulation of Shade Avoidance Under Low‐Blue‐Light by MTA in Soybean
Source: Adv Sci (Weinh). 2024 Dec 12;12(5):2410334. doi: 10.1002/advs.202410334 (PMC11791948; doi:10.1002/advs.202410334)
Supplement: Supplementary file 1 — Supporting Information [file ADVS-12-2410334-s003.docx]

Supporting Information

Regulation of shade avoidance under low-blue-light by MTA in soybean

Liya Zhang^1,*^, Jun Liu^1,*^, Jiaqi Chen^1^, Yanyan Zhang^1^, Chao Qin^1^, Xiangguang Lyu^1^, Zhuang Li^1^, Ronghuan Ji^1^, Bin Liu^1🖂^, Hongyu Li^1🖂^, Tao Zhao^1🖂^


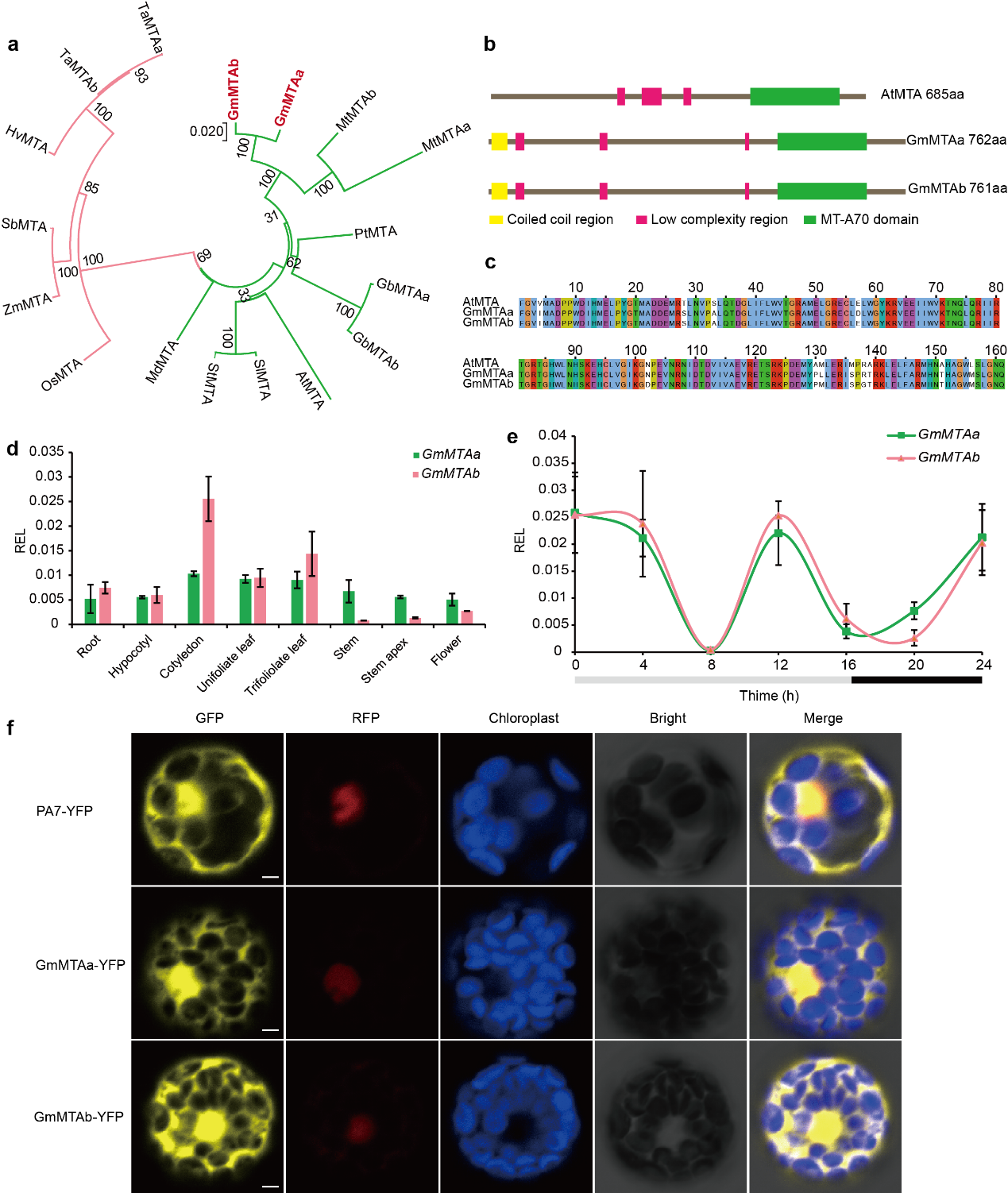


**Figure S1 *Glycine max* mRNA adenosine methylase A (GmMTAs), two key components of the modification complex in soybean.** a, Phylogenetic tree of GmMTAs proteins with other plant MTA proteins using the neighbor-joining method by the MEGA5. Nomenclatures are as follows: *At*, *Arabidopsis thaliana*; *Gm*, *Glycine max*; *Mt*, *Medicago truncatula*; *Pt*, *Populus trichocarpa*; *Gb*, Gossypium barbadense; *Sl*, *Solanum lycopersicum*; *St*, *Solanum tuberosum*; *Md*, *Malus domestica*; *Os*, Oryza sativa; *Zm*, Zea mays; *Sb*, Sorghum bicolor; *Hv*, *Hordeum vulgare r1*; *Ta*, *Triticum aestivum*. The green line and the pink line represent dicotyledon plants monocotyledon plants, respectively. GmMTAa and GmMTAb are marked with red letters. b, Protein domain of soybean and *Arabidopsis* MTA proteins using SMART web pages. The yellow boxes, pink boxes and green boxes represent Coiled coil region, Low complexity region and MT-A70 domain, respectively. c, Alignment of the amino acid sequence of MT-A70 domain of soybean and *Arabidopsis*. d, Transcriptional analysis of GmMTAs in different tissues of soybean cultivar Williams 82 by qRT–PCR. *GmActin* was used as an internal control. Data are shown as means ± SD (n = 3). REL, relative expression level. e, Time course transcriptional analysis of *GmMTAs* under long day conditions (16 h light/8 h dark). f, Subcellular localization of GmMTAs proteins in *Arabidopsis* mesophyll protoplasts. PA7-YFP was used as a control. Scale bars, 5 μm.


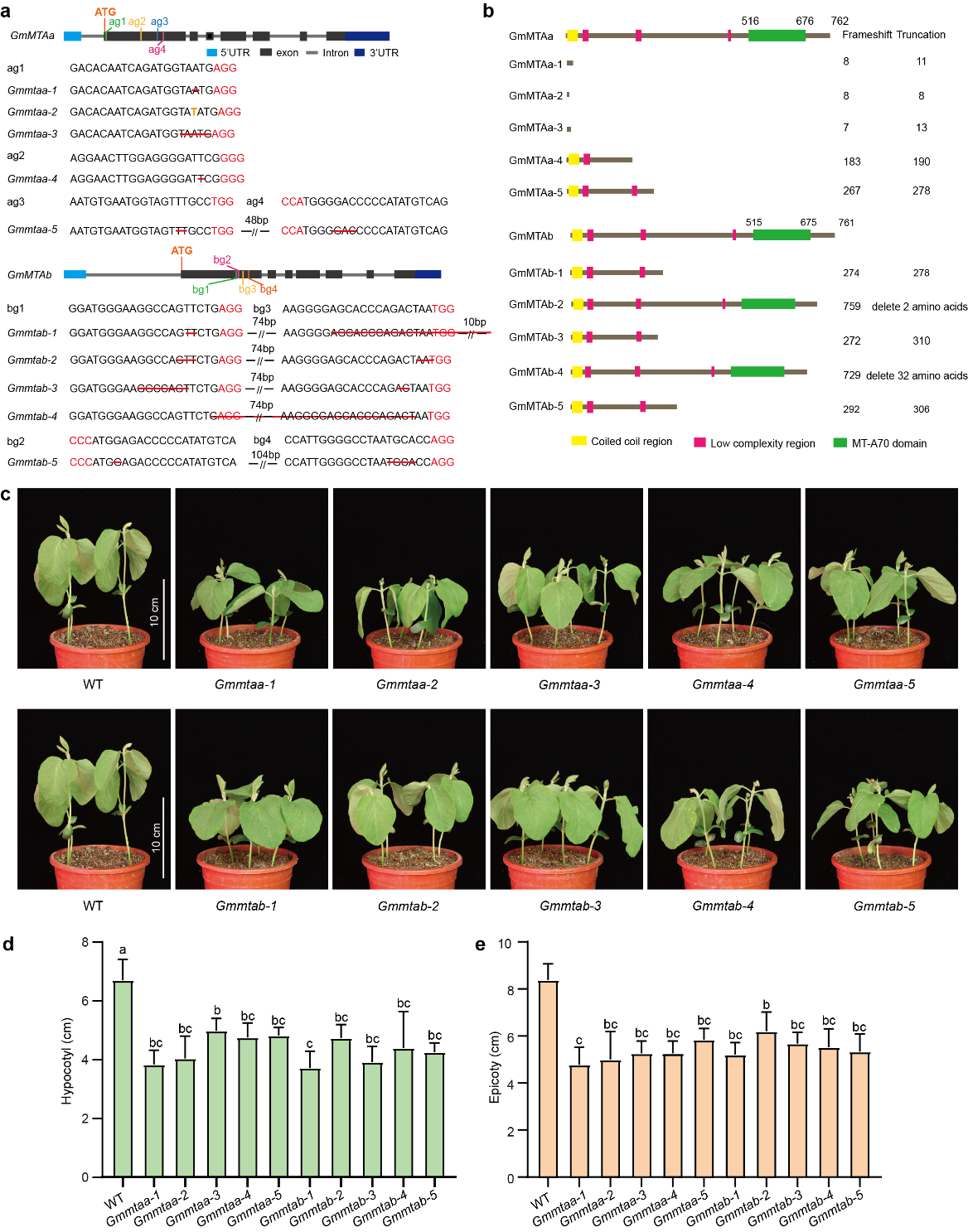


**Figure S2** **Plant height phenotype of *Gmmtaa and Gmmtab* mutants under long**-**day conditions.** a, Schematic diagram showing the location of mutated sites targeted by gRNAs on the *GmMTAa* and *GmMTAb* genome. The ag and bg letters represent the conserved gRNA target site of *GmMTAa* and *GmMTAb* genes, respectively. Nucleotides in red indicate the PAM. Light blue boxes indicate 5’ untranslated regions (UTRs); Dark grey boxes denote exons; Light gray lines are introns; Dark blue boxes represent 3’ untranslated regions (UTRs). The red horizontal lines on the letters represents the nucleotide deletions in the *GmMTA*s genes. b, Schematic diagram of the mutant GmMTAs proteins in *Gmmtaa* and *Gmmtab* single plants. The numbers indicate the position of the amino acid frameshift and truncation. c, Representative images of *Gmmtas* single plants grown in long day conditions. Scale bar, 10 cm. **d,e,** Statistical analysis of the hypocotyl (**d**) and epicotyl (**e**) of the indicated lines as in (**c**). Data are shown as means ± SD (n ≥ 6) with Ordinary one-way ANOVA, followed by a Tukey multiple comparisons posttest (*P*<0.05).


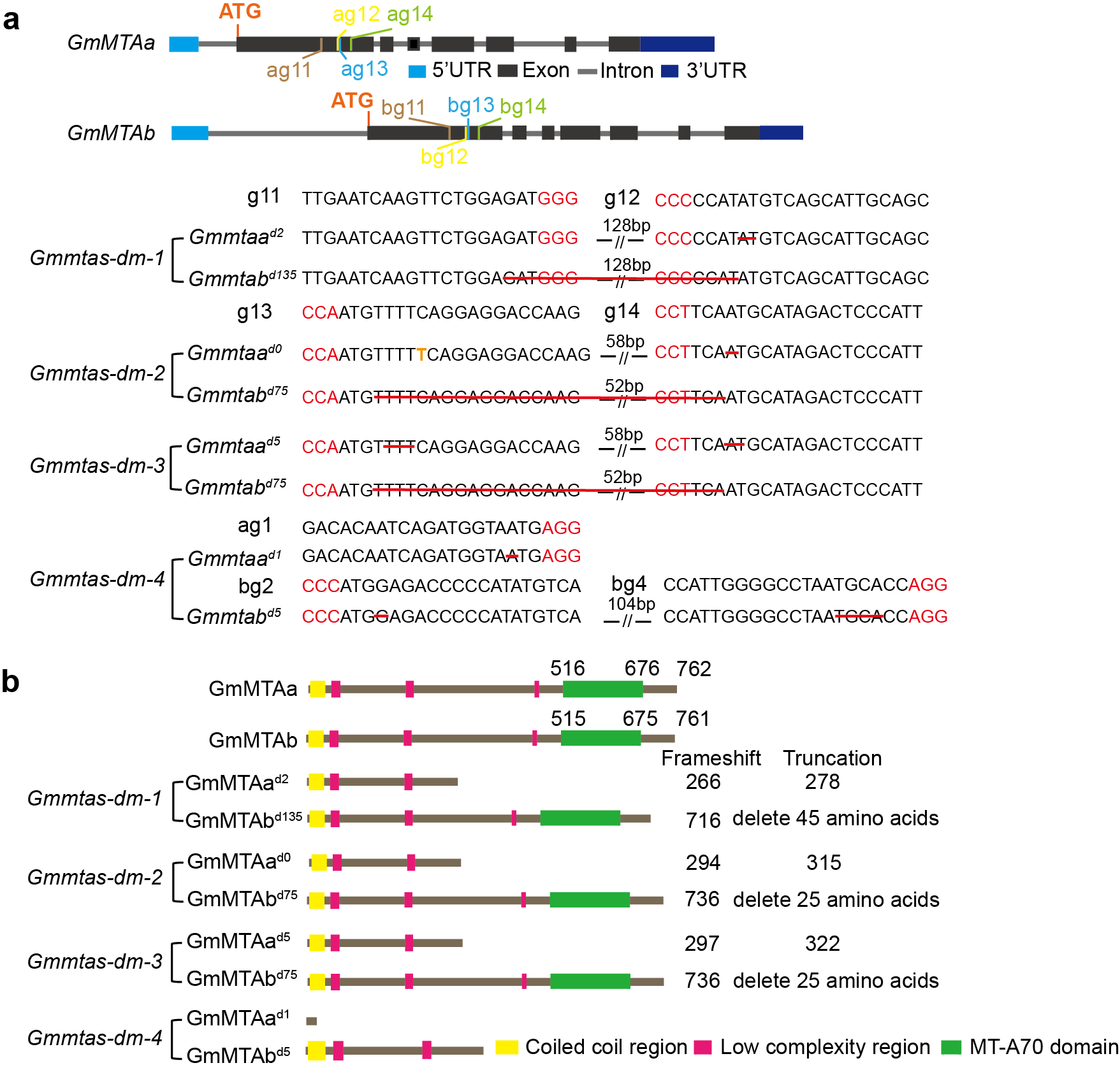


**Figure S3 The genomic structures and protein domain of *Gmmtas* double mutants. a**, Schematic diagram showing the genomic structures of *GmMTAa* and *GmMTAb* genes. The ag and bg letters represent the conserved gRNA target site of *GmMTAa* and *GmMTAb* genes, respectively. Nucleotides in red indicate the PAM. Light blue boxes indicate 5’ untranslated regions (UTRs); Dark grey boxes denote exons; Light gray lines are introns; Dark blue boxes represent 3’ untranslated regions (UTRs). The red horizontal lines on the letters and the bold orange letter represent the nucleotide deletions and insertion in the *GmMTA*s genes, respectively. b, Schematic diagram of the mutant GmMTAs protein in *Gmmtas* double mutants. The numbers indicate the position of the amino acid frameshift and truncation.


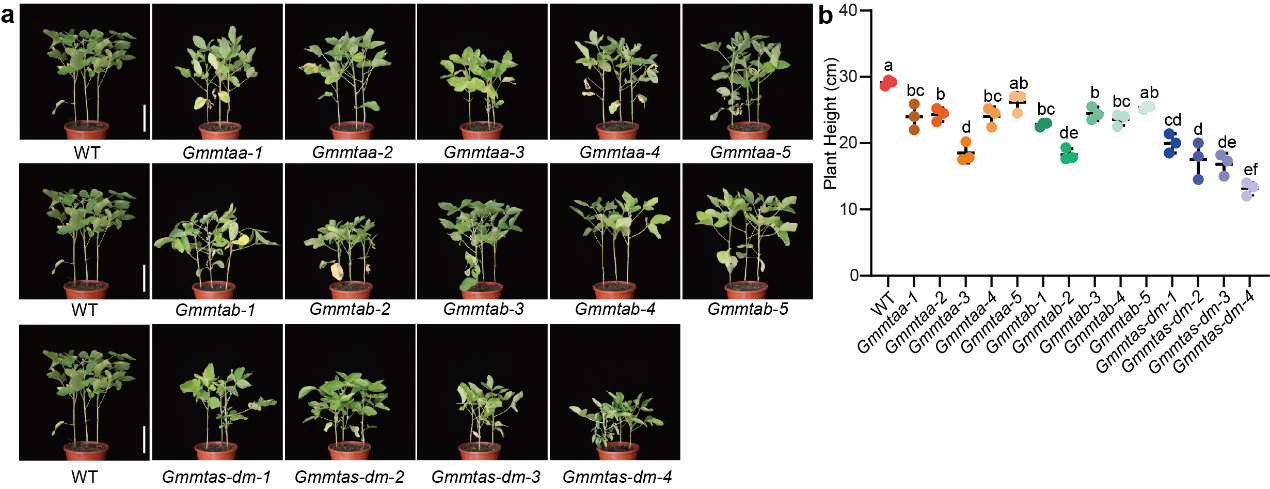


**Figure S4 Plant height phenotype of *Gmmtas* single and double mutants under natural long**-**day conditions.** **a,** Representative images of *Gmmtas* single and double mutants grown under natural long day conditions. Scale bar, 10 cm. **b,** Statistical analysis of the plant height of the indicated lines as in (**a**). Data are shown as means ± SD (n = 3) with Ordinary one-way ANOVA, followed by a Tukey multiple comparisons posttest (*P*<0.05).


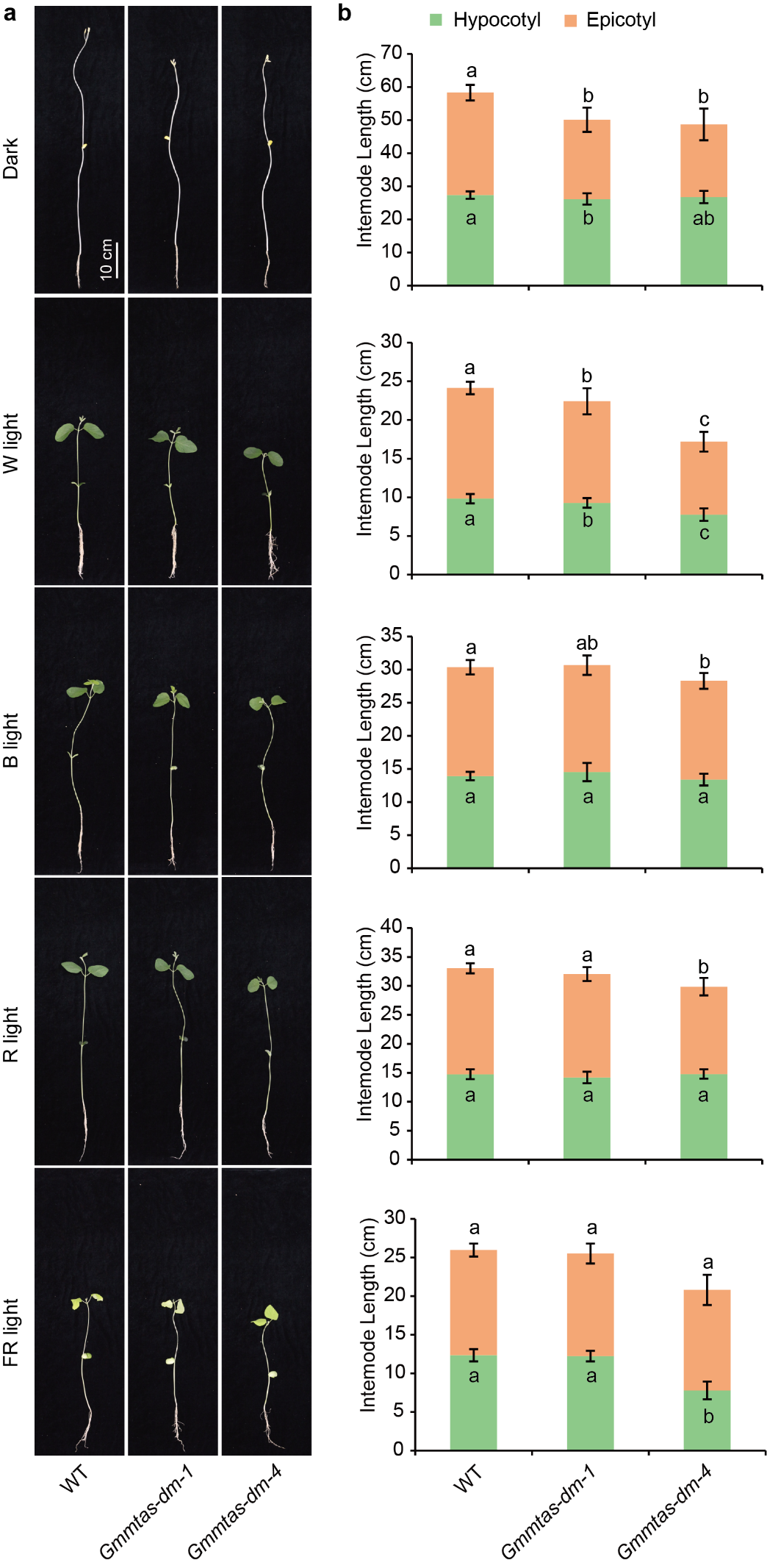


**Figure S5 The elongated seedling phenotype of Gmmtas-dm mutants.** **a,** Photographs with indicated lines grown in the dark and under continuous White (W) light (100 µmol·m^−2^·s^−1^), Blue (B) light (50 µmol·m^−2^·s^−1^), Red (R) light (50 µmol·m^−2^·s^−1^) and Far Red (FR) (30 µmol·m^−2^·s^−1^) at 25–26°C for 10 days. Scale bar, 10 cm. **b,** Statistics of hypocotyl and epicotyl lengths of the seedlings shown in (**a**) Data are shown as means ± SD of at least 8 seedlings. Significant differences between any two groups were evaluated by Ordinary one-way ANOVA, followed by a Tukey multiple comparisons posttest (*P*<0.05).


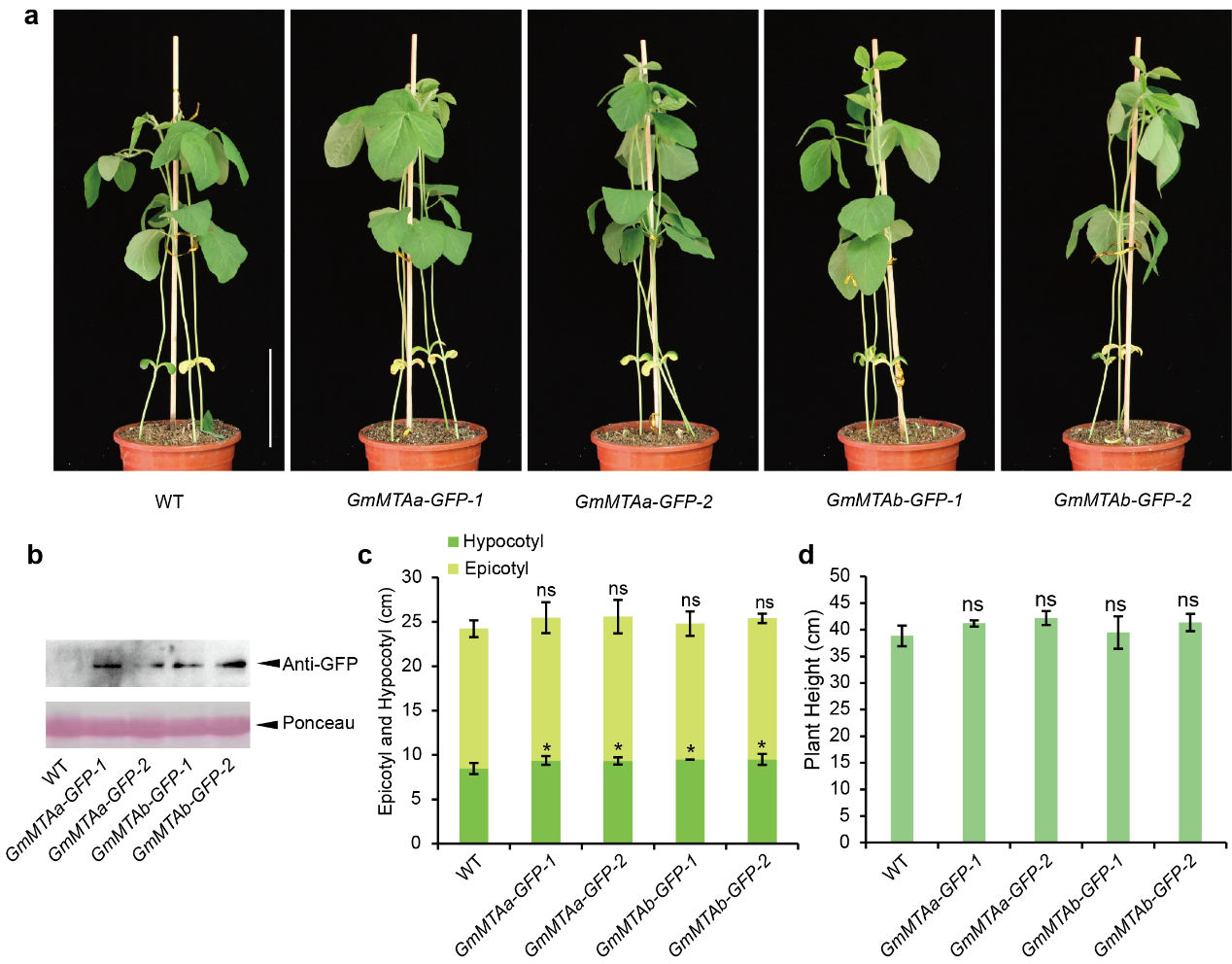


**Figure S6 Plant height phenotype of the overexpression lines of *GmMTAs*.** **a,** Representative images of the overexpression lines of *GmMTAs* plants grown in long day conditions for 20 days. Scale bar, 10 cm. **b,** Immunoblots show the expression of GmMTAa-GFP and GmMTAb-GFP fusion proteins in the transgenic plants using the anti-GFP antibody. WT sample was used as negative control. The ponceau was used as the loading control. **c,** Statistical analysis of the hypocotyl and epicotyl, as well as plant height (**d**) of the indicated lines as in (**a**). Data are shown as means ± SD (n ≥ 3) by Student’s t-tests (**P*<0.05).


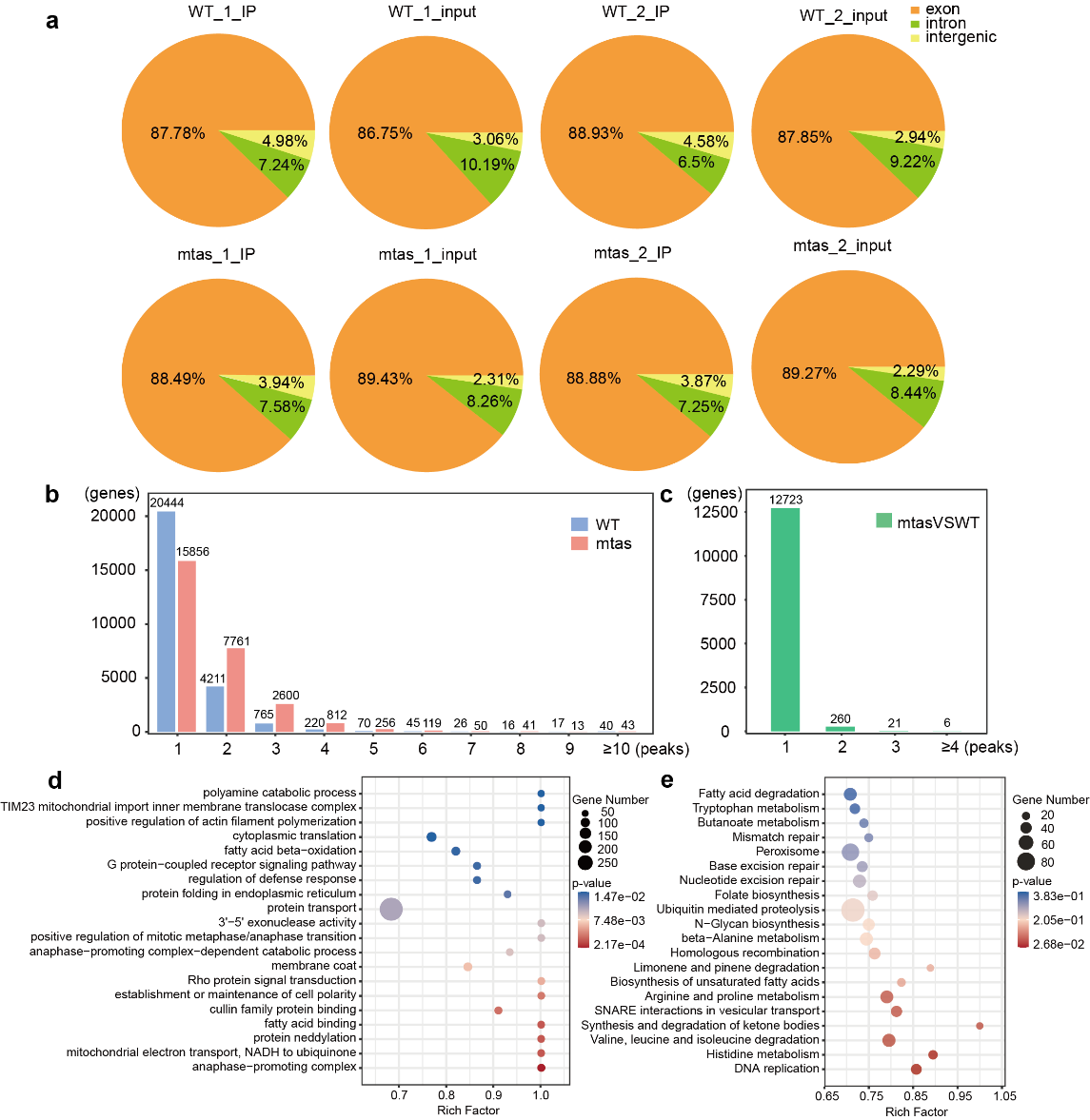


**Figure S7 *GmMTAs* mediate epitranscriptomes in soybean.** **a,** The distribution of reads in different regions of the reference genome (*Glycine max Wm82.a2.v1*) in two biological replicated WT and *Gmmtas-dm-4* seedlings. **b,** The number of genes with methylated m^6^A peaks in WT and *Gmmtas-dm-4*. **c,** The histogram of the hypomethylated m^6^A peaks identified in *Gmmtas-dm-4*. **d, e,** GO (**d**) and KEGG (**e**) analysis of the hypomethylated m^6^A containing genes in *Gmmtas-dm-4*.


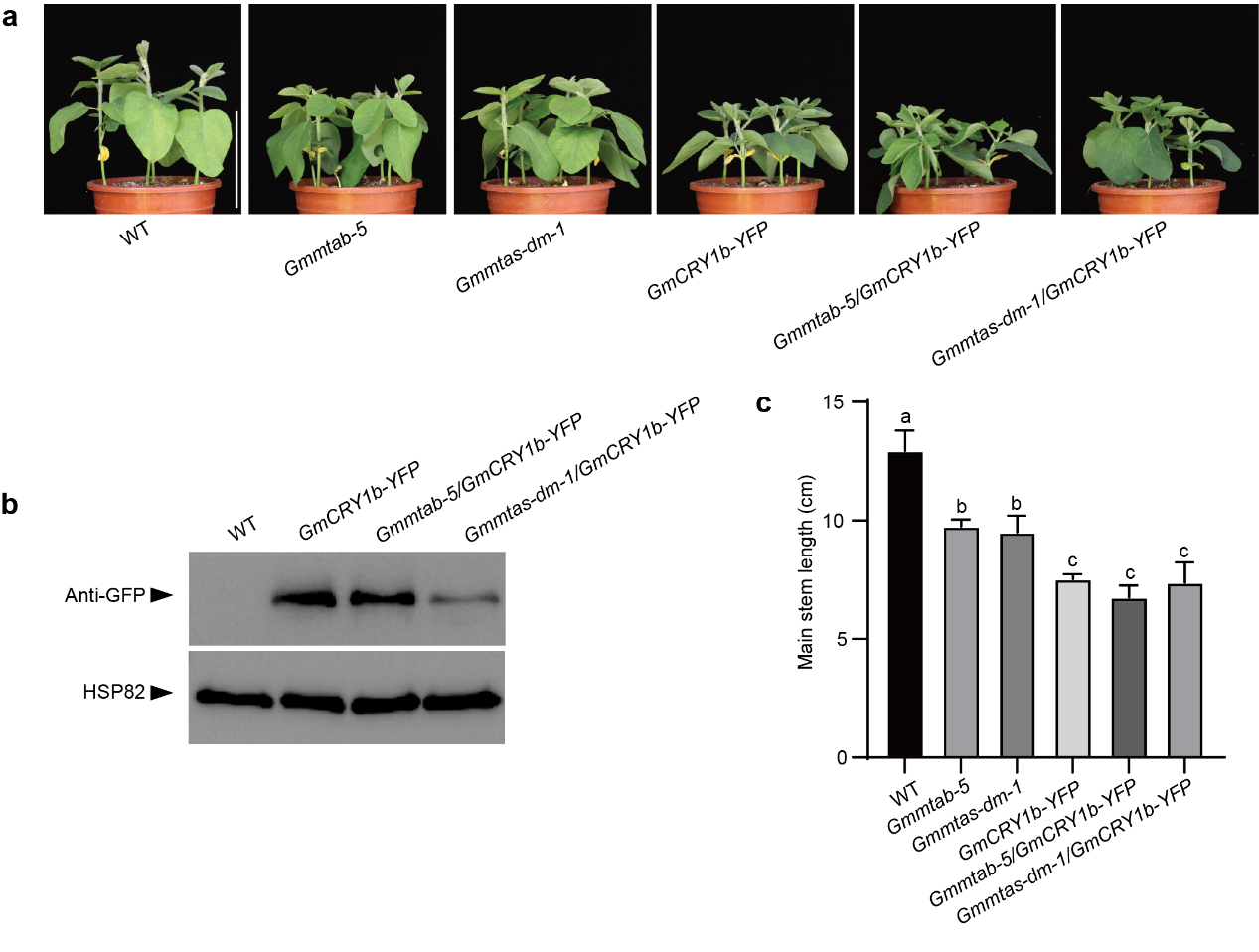


**Figure S8 *GmMTAs* regulates plant height by *GmCRY1b* in soybean.** **a,** The hybrid material of *Gmmtas* mutants and *GmCRY1b*-*YFP* showed the phenotype of *GmCRY1b*-*YFP*. **b,** Immunoblots show the expression of GmCRY1b-YFP fusion proteins in the transgenic plants using the anti-GFP antibody. WT sample was used as negative control. HSP82 was used as the loading control. **c,** Statistical analysis of the plant height of the indicated lines as in (**a**). Data are shown as means ± SD (n = 4) by Ordinary one-way ANOVA, followed by a Tukey multiple comparisons posttest (*P*<0.05).


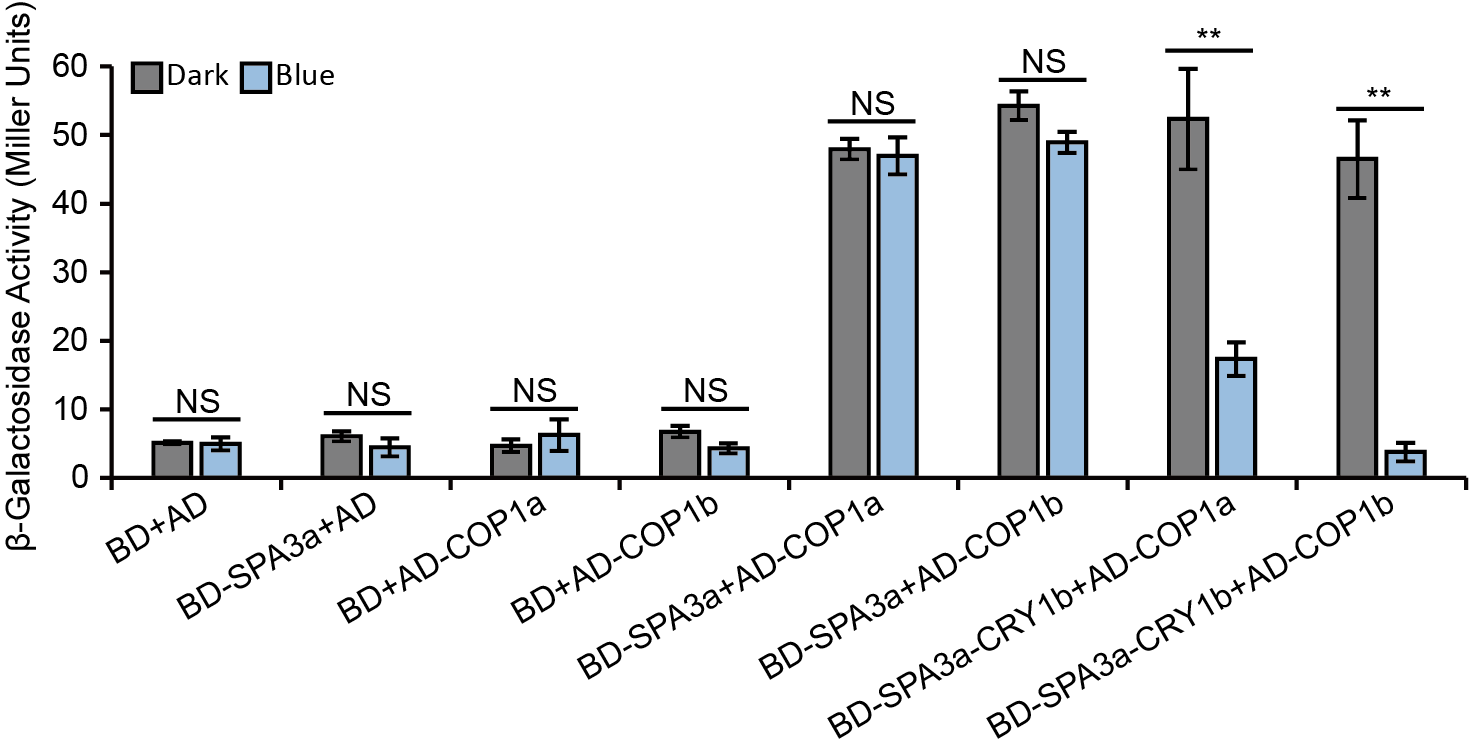


**Figure S9 GmCRY1b interact with GmSPA3a to inhibit the GmSPA3a-GmCOP1a/GmCOP1b interaction under** **continuous blue light (30 μmol·m^-2^·s^-1^).** Yeast cells (AH109) expressing the indicated proteins were cultured in SD medium (−Leu/-Met/−Trp/+Asp) and then transferred into 8 mL YPDA medium until OD600= 0.7-1.0 under dark and continuous blue light (30 μmol·m^−2^·s^−1^), respectively, followed by β-galactosidase assays.


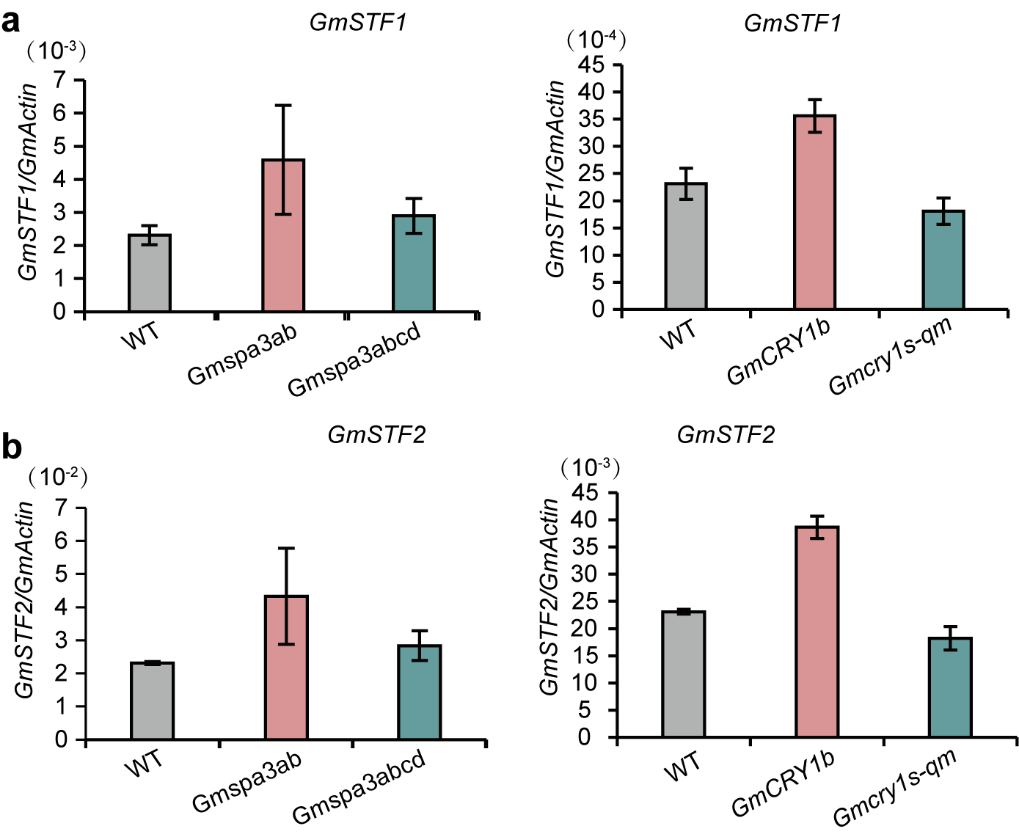


**Figure S10 Analysis of the *GmSPAs* and *GmCRY1s* effect on the expression of *GmSTFs* genes. a,** **b,** The expression levels of *GmSTF1* (**a**) and *GmSTF2* (**b**) in *Gmspas* and *Gmcry1s-qm* mutants. The young unifoliolate leaves were collected at 7 days under continuous white light. Each sample was analyzed in triplicate.


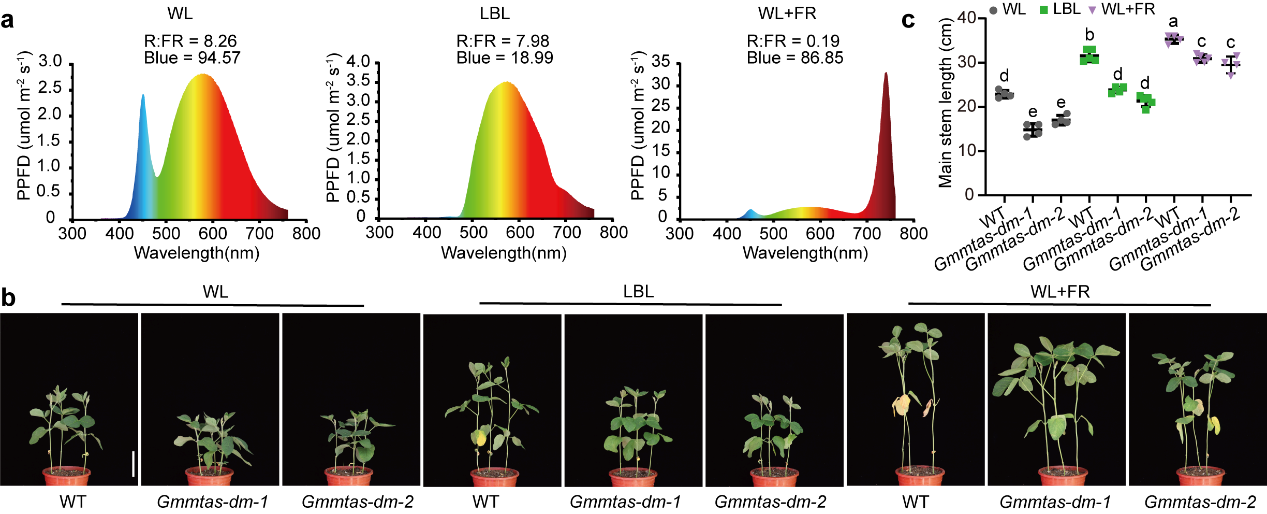


**Figure S11. *GmMTAs* regulates shade avoidance induced by low blue light. a,** Light spectral compositions of white light (WL), low blue light (LBL), WL plus far-red light (WL+FR). Low R:FR was achieved by supplementing far-red light to WL; LBL was achieved by filtering WL through two layers of yellow filters. The light quality and intensity of WL, LBL and WL+LBL were all 500 μmol·m^−2^·s^−1^. **b,** Representative images of the indicated lines grown under WL, LBL and WL+FR conditions. 10 days seedlings were treated with the indicated light regimes in (**a**) under long day conditions for 10 days. Scale bar, 10 cm. **c,** Statistical analysis of the plant height of each line in (**b**). Data are shown as means ± SD (n ≥ 4). The letters indicate significant differences with Ordinary one-way ANOVA, followed by a Tukey multiple comparisons posttest (*P*<0.05).
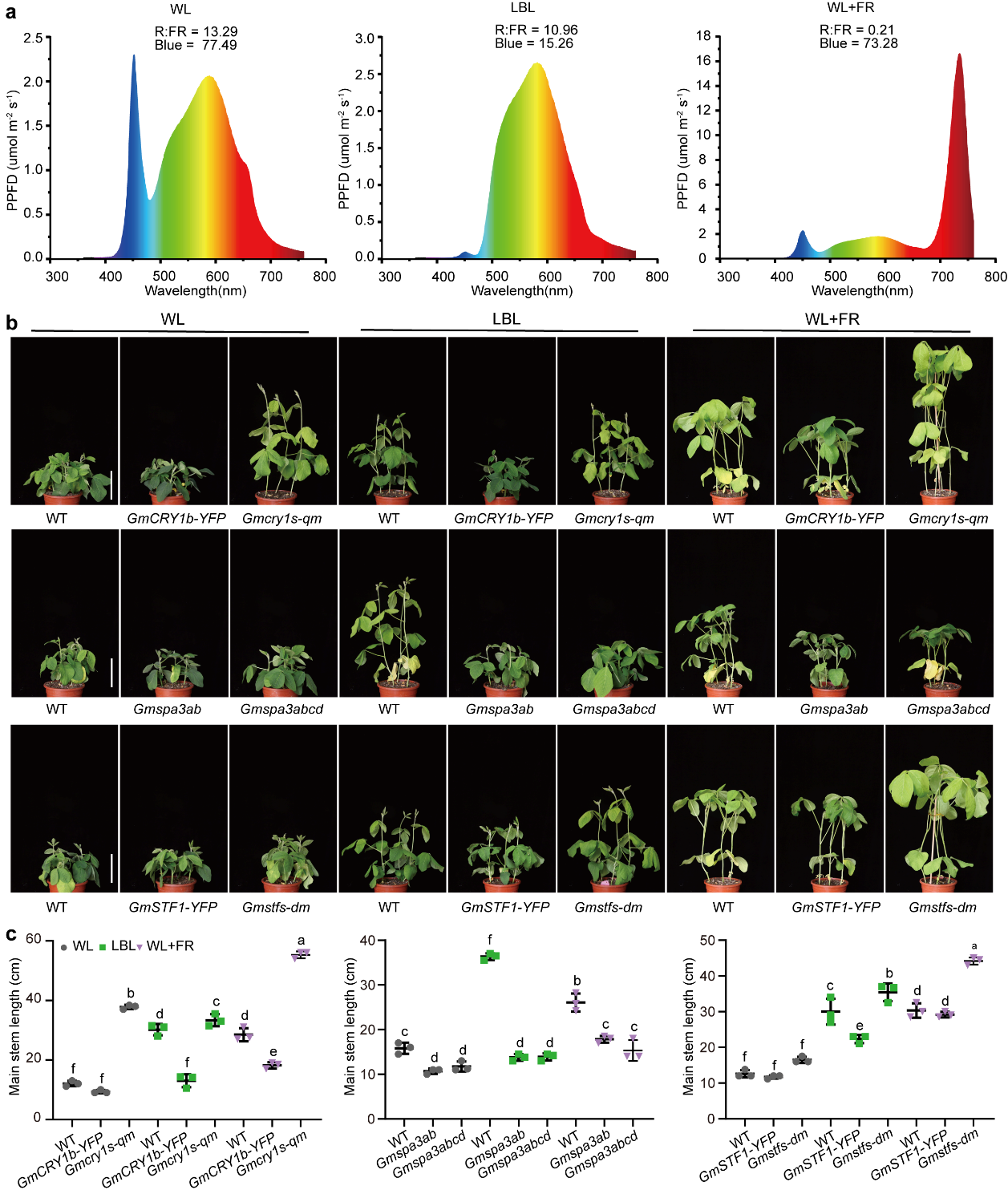


**Figure S12 *GmCRY1b*-*YFP*, *Gmspa3ab*, *Gmspa3abcd* and *GmSTF1-YFP* exhibited good shade tolerance under LBL conditions.** **a,** Light spectral compositions of white light (WL), low blue light (LBL), WL plus far-red light (WL+FR). Low R:FR was achieved by supplementing far-red light to WL; LBL was achieved by filtering WL through two layers of yellow filters. The light quality and intensity of WL, LBL and WL+LBL were all 350 μmol·m^−2^·s^−1^. **b,** Representative images of the indicated lines grown under WL, LBL and WL+FR conditions. 12 days seedlings were treated with the indicated light regimes in (**a**) under long day conditions for 12 days. Scale bar, 10 cm. **c** Statistical analysis of the plant height of each line in (**b**). Data are shown as means ± SD (n = 3). The letters indicate significant differences with Ordinary one-way ANOVA, followed by a Tukey multiple comparisons posttest (*P*<0.05).


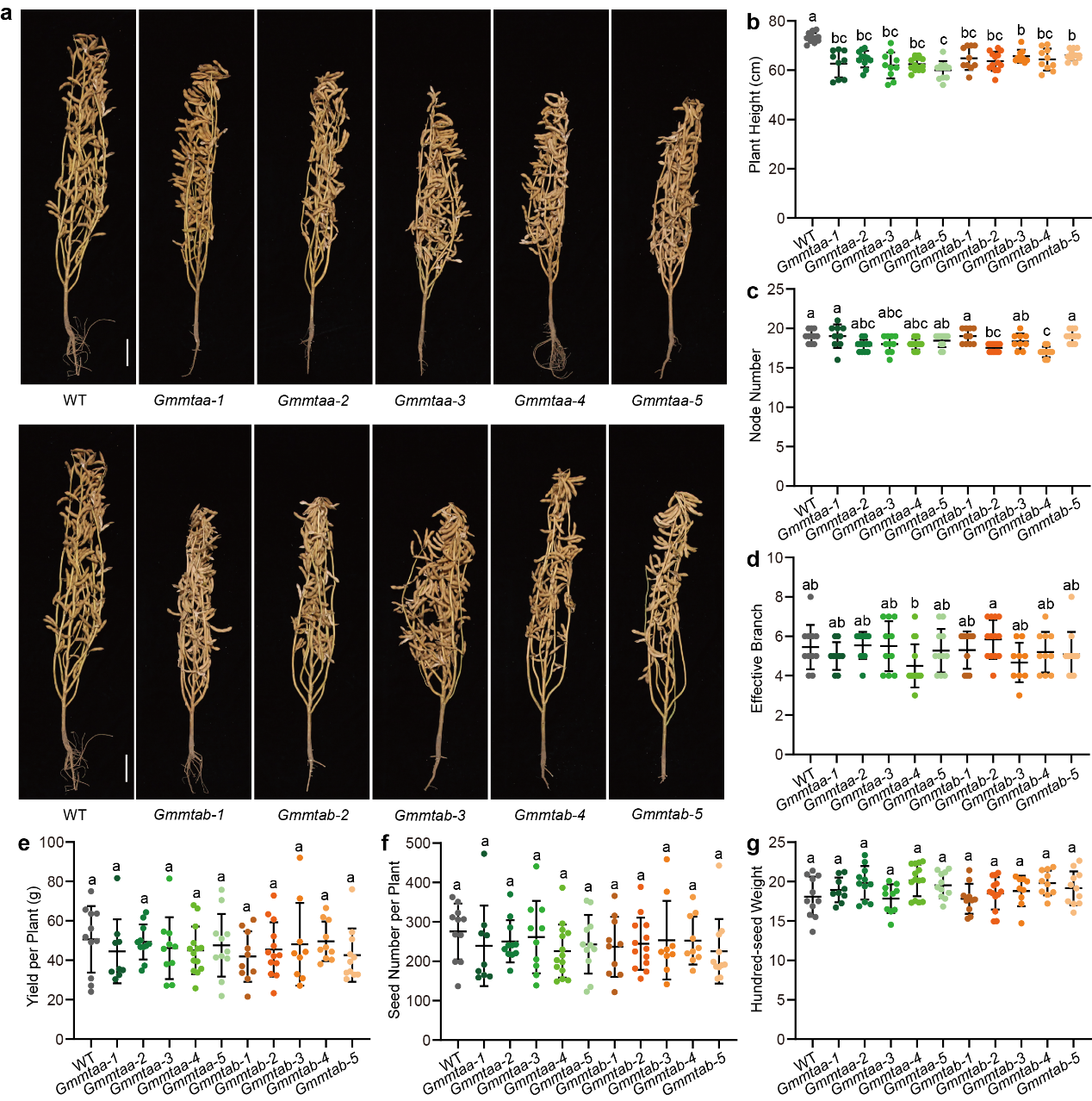


**Figure S13 CRISPR/Cas9-engineered single mutations in *GmMTAs* confer reduced plant height phenotype.** **a,** Representative images of *GmMTAs* single plants grown in Yihai, Beijing in the summer of 2022, China (40.1°N, 116.7°E). Scale bar, 10 cm. **b,** Statistical analysis of the agronomic traits of the indicated lines as in (**a**). Data are shown as means ± SD (n ≥ 9) with significant difference by Ordinary one-way ANOVA, followed by a Tukey multiple comparisons posttest (*P*<0.05).


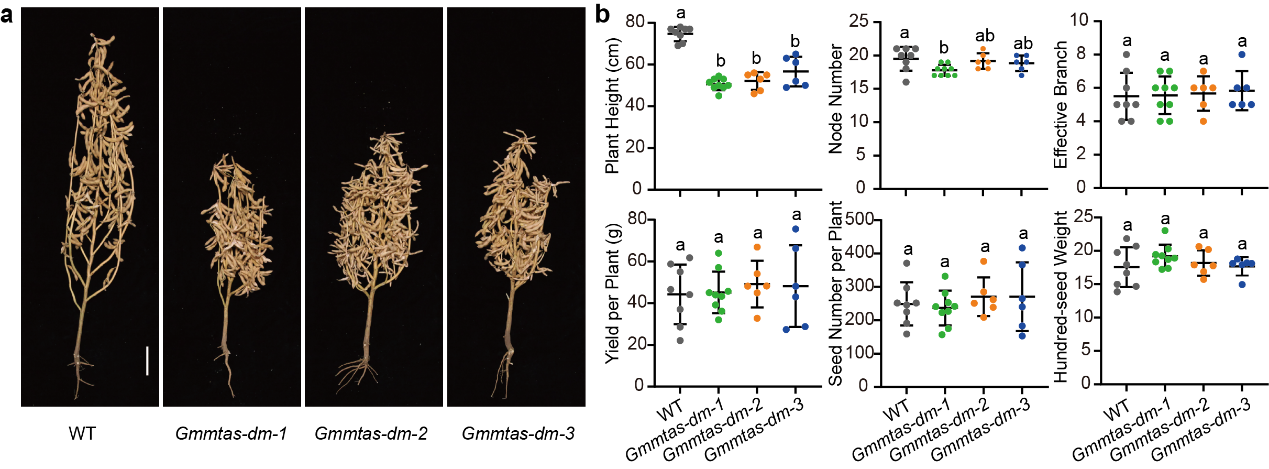


**Figure S14 CRISPR/Cas9-engineered double mutations in *GmMTAs* confer reduced plant height phenotype. a,** Representative images of *GmMTAs* single plants grown in Yihai, Beijing in the summer of 2022, China (40.1°N, 116.7°E). Scale bar, 10 cm. **b,** Statistical analysis of the agronomic traits of the indicated lines as in (**a**). Data are shown as means ± SD (n ≥ 6) with significant difference by Ordinary one-way ANOVA, followed by a Tukey multiple comparisons posttest (*P*<0.05).


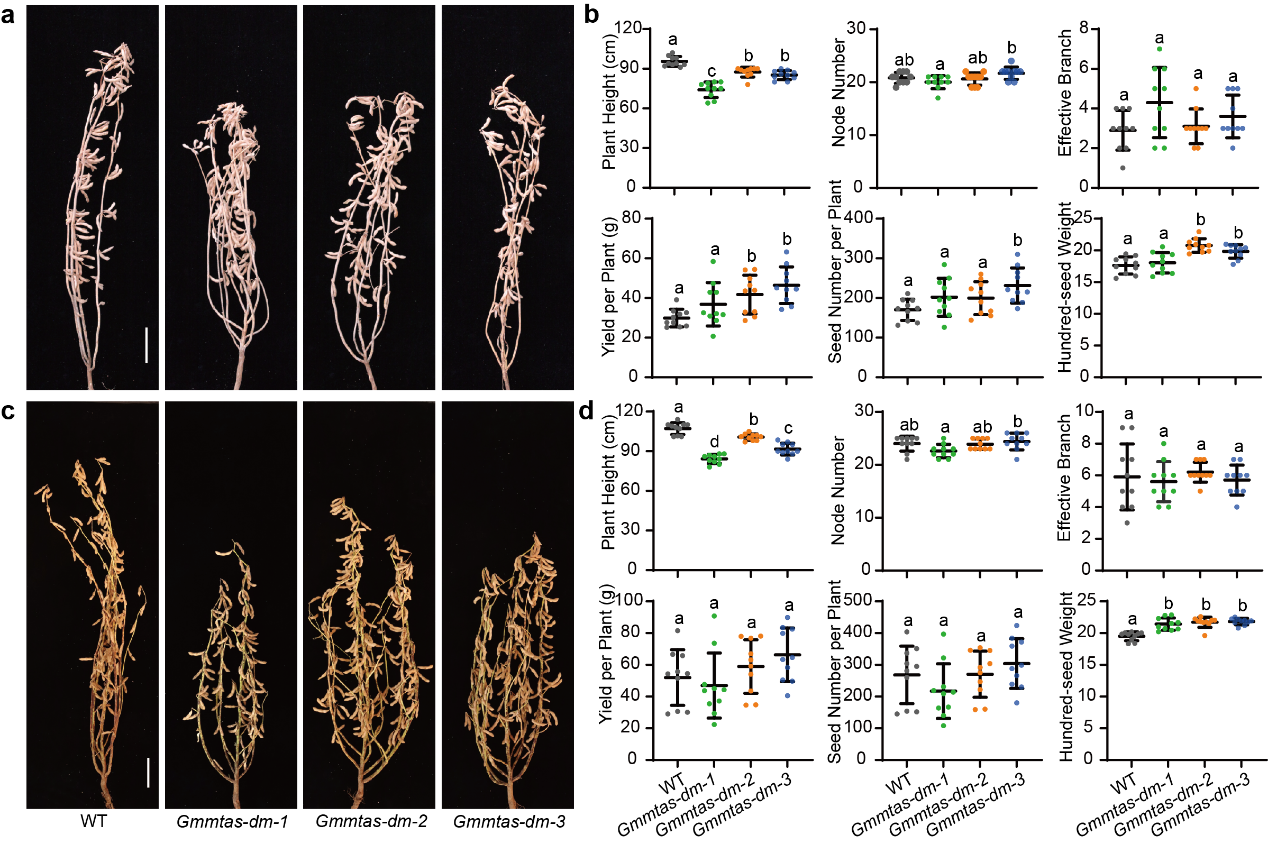


**Figure S15 CRISPR/Cas9-engineered double mutations in *GmMTAs* confer reduced plant height phenotype**. **a,** Representative images of *GmMTAs* double plants grown in Shunyi, Beijing in the summer of 2023, China (40.2°N, 116.6°E). Scale bar, 10 cm. **b,** Statistical analysis of the agronomic traits of the indicated lines as in (**a**). Data are shown as means ± SD (n = 10) with significant difference by Ordinary one-way ANOVA, followed by a Tukey multiple comparisons posttest (*P*<0.05). **c,** Representative images of *GmMTAs* double plants grown in Changchun, Jilin in the spring of 2023, China (43.8°N, 125.4°E). **d,** Statistical analysis of the agronomic traits of the indicated lines as in (**c**). Data are shown as means ± SD (n = 10) with significant difference by Ordinary one-way ANOVA, followed by a Tukey multiple comparisons posttest (*P*<0.05).
